# Whole transcriptome profiling of patient-derived xenograft models as a tool to identify both tumor and stromal specific biomarkers

**DOI:** 10.18632/oncotarget.8014

**Published:** 2016-03-09

**Authors:** James R. Bradford, Mark Wappett, Garry Beran, Armelle Logie, Oona Delpuech, Henry Brown, Joanna Boros, Nicola J. Camp, Robert McEwen, Anne Marie Mazzola, Celina D'Cruz, Simon T. Barry

**Affiliations:** ^1^ Department of Oncology and Metabolism, University of Sheffield, Sheffield, South Yorkshire, UK; ^2^ Oncology iMED, AstraZeneca Pharmaceuticals, Alderley Park, Cheshire, UK; ^3^ Department of Internal Medicine and Huntsman Cancer Institute, University of Utah, Salt Lake City, Utah, USA; ^4^ Oncology iMED, AstraZeneca Pharmaceuticals, Gatehouse Park, Massachusetts, USA

**Keywords:** patient-derived xenograft, RNA-Seq, tumor stroma, biomarker discovery, pre-clinical research

## Abstract

The tumor microenvironment is emerging as a key regulator of cancer growth and progression, however the exact mechanisms of interaction with the tumor are poorly understood. Whilst the majority of genomic profiling efforts thus far have focused on the tumor, here we investigate RNA-Seq as a hypothesis-free tool to generate independent tumor and stromal biomarkers, and explore tumor-stroma interactions by exploiting the human-murine compartment specificity of patient-derived xenografts (PDX).

Across a pan-cancer cohort of 79 PDX models, we determine that mouse stroma can be separated into distinct clusters, each corresponding to a specific stromal cell type. This implies heterogeneous recruitment of mouse stroma to the xenograft independent of tumor type. We then generate cross-species expression networks to recapitulate a known association between tumor epithelial cells and fibroblast activation, and propose a potentially novel relationship between two hypoxia-associated genes, human *MIF* and mouse *Ddx6*. Assessment of disease subtype also reveals *MMP12* as a putative stromal marker of triple-negative breast cancer. Finally, we establish that our ability to dissect recruited stroma from trans-differentiated tumor cells is crucial to identifying stem-like poor-prognosis signatures in the tumor compartment.

In conclusion, RNA-Seq is a powerful, cost-effective solution to global analysis of human tumor and mouse stroma simultaneously, providing new insights into mouse stromal heterogeneity and compartment-specific disease markers that are otherwise overlooked by alternative technologies. The study represents the first comprehensive analysis of its kind across multiple PDX models, and supports adoption of the approach in pre-clinical drug efficacy studies, and compartment-specific biomarker discovery.

## INTRODUCTION

The tumor stroma comprises of numerous cell types including endothelial cells, cancer-associated fibroblasts (CAFs), mesenchymal stem cells, and immune cells such as lymphocytes and tumor-associated macrophages. It plays a critical role in supporting cancer growth and metastasis [1], and is therefore emerging as rich source of targets for anti-cancer therapy. However, we have a poor understanding of the interactions between tumor and stroma. A typical solid tumor tissue sample consists of both components, and such sample heterogeneity can have significant influence on the biological interpretation of genomic profiling studies [2]. Furthermore, attempts to separate tumor from stroma are hampered by the requirement for specialist techniques such as laser capture micro-dissection, with small amounts of tumor cell contamination possible.

Patient-derived tumor xenograft (PDX) models are generated when fresh tumor tissue obtained directly from patients is implanted subcutaneously or orthotopically into immune-deficient mice. As such, they maintain the principal histological, clinical and molecular characteristics of the original patients' tumors while remaining biologically stable when passaged in mice [3–5]. Since PDX models more closely resemble and recapitulate tumor growth in humans than standard *in vitro* cell line or cell line xenograft approaches, they remain key experimental platforms for pre-clinical drug development.

Recent studies have shown that human and mouse transcription can be accurately differentiated in PDX models using RNA-Seq [6–7], removing the need for manipulation of the RNA population, customised sequencing protocols, or prior knowledge of the species component ratio. Moreover, the known transcriptional response to drugs targeting the stroma can be accurately recapitulated in both human tumor and mouse stroma [6]. The high specificity of the *in silico* read disambiguation approach means that gene expression in the human component is quantified almost exclusively from tumor RNA, particularly in later passages where the original patient stroma has been replaced by mouse stroma. Thus PDX transcriptome data provide a unique opportunity for the simultaneous study of both tumor and stromal specific signals *in vivo*. Consequently, several studies have adopted the approach albeit restricted to only a small number of xenograft models or specific cancer type [8–9].

In this paper, we build significantly on these early studies by using RNA-Seq to profile the baseline human and mouse transcriptome of 79 PDX models representing multiple cancer types. By doing so, we assess mouse stromal heterogeneity, generate hypotheses on the relationship between mouse stroma and human tumor, and identify both tumor and stromal specific markers of disease subtype. To our knowledge, this represents the first comprehensive analysis of both species components of PDX models simultaneously across such a large cohort. As such, the dataset should provide a key platform for gaining additional insights into PDX tumor and stroma processes, and interpreting pre-clinical efficacy studies.

## RESULTS

**Figure 1:**
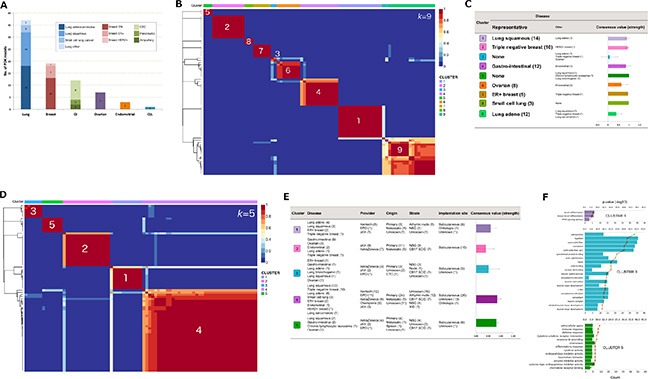
Application of non-negative matrix factorization (NMF) to optimal clustering of human and mouse gene expression (**A**) disease representation across the 79 PDX models. (**B**) consensus matrix at *k* = 9 for the human transcriptome. (**C**) contributing cancer types and mean consensus value of each human cluster. “Representative” disease indicates the majority cancer type in the cluster, and numbers of models are given in brackets. Mean consensus value was computed from 200 runs of NMF. (**D**) consensus matrix at *k* = 9 for the mouse transcriptome. (**E**) meta-data breakdown in each mouse cluster and mean consensus value. (**F**) functional enrichment of the meta-genes driving the mouse clustering. Only clusters with significant functional enrichment (FDR < 0.05) are shown.

79 PDX models from five different providers covering seven cancer types were used in the study (Figure 1A, Table S1) with the majority of models representing lung (37 models) and breast (19 models). A mean of 47,906,117 human and 6,612,995 mouse reads were uniquely mapped to a concatenated human and mouse genome (Figure S1A, Table S2), corresponding to a human component of ∼88% in each sample (Figure S1B, Table S2). We have previously demonstrated that low coverage (1.3–2.0 M reads) was sufficient to deliver accurate detection and measurement of mouse gene expression [6]. In this study we generated a coverage of 5–10 M reads, which was considered sufficient to generate a robust data set. Expression between technical and biological replicates showed strong correlation across both human (Figure S2A–S2C) and mouse (Figure S2D–S2F) genes. Predicted stromal and immune cell content using ESTIMATE [10] indicated a clear differentiation between the human and mouse components, and > 97% tumor purity in the human component of 76/79 samples (Figure S3, Table S3). As a proxy for the quantity of original patient stroma in each PDX sample, human expression levels of two CAF markers that are rarely expressed by tumor epithelial cells, fibroblast activation protein alpha (*FAP*) and chondroitin sulfate proteoglycan 4 (*CSPG4*), were assessed. 22/79 samples showed evidence of patient stroma retention at a low stringency CAF marker expression threshold (*FAP* or *CSPG4* log_2_ FPKM > 2.0; Table S3) and therefore flagged as potential confounders in analyses of the human component. Of these, only 11 samples expressed high levels of either marker (log_2_ FPKM > 4.0), and overall results suggest human and mouse transcriptional profiles reflect highly enriched human tumor and mouse stroma cell populations respectively in the majority of samples.

### Mouse stroma heterogeneity is primarily driven by dominant cell type

We applied non-negative matrix factorization (NMF) to cluster 14173 and 3933 of the most highly expressed (human: FPKM > 10, mouse: FPKM > 2 in at least one sample) and variable (coefficient of variation > 0.20) genes across human and mouse respectively, and test whether gene expression signatures exist in the mouse component allowing separation into distinct subtypes. Stable clusters were achieved at *k* = 9 (human; Figure 1B) and *k* = 5 (mouse; Figure 1D) where *k* denotes the number of clusters and values selected according to the procedure outlined in *Methods*. Model-to-cluster mappings for both human and mouse are given in Tables S4A and S4B respectively, and genes deemed as key drivers of the clustering (meta-genes) are listed in Tables S5A (human) and S5B (mouse).

Human clusters were more strongly associated with cancer type (*p* < 2.20E-16 by Chi-squared test; Figure 1C) than the mouse clusters (*p* = 1.07E–5; Figure 1E). 8/15 and 7/11 tumors in human clusters 1 and 2 respectively expressed CAF markers *FAP*/*CSPG4*, which may suggest a propensity for some lung squamous and triple-negative breast cancers (TNBC) to retain patient stroma.

Functional enrichment analysis using Toppgene [11] revealed that 3/5 mouse clusters encompassed strong functional themes associated with fat (cluster 1), muscle (cluster 3) and immune cells (cluster 5; Figure 1F, Table S6) corresponding to strong overlap with adipocyte (*p* = 1.70E–16), embryonic stem (*p* = 2.14E–37), and myeloid (*p* = 8.68E–28) cell type signatures respectively. Cluster 2 was primarily driven by *Col10a1* expression (relative contribution to meta-gene = 0.89; Table S5B), a potential marker of CD10+ tumor stromal cells [12], and cluster 4 showed some enrichment for mesenchymal stem cell markers (*p* = 1.01E–08).

The mean number of mapped mouse reads (Figure S4A) or proportion of mouse component (Figure S4B) was not significantly different between mouse clusters. Notably, cluster 5 included samples from model HOXF060 with the largest mouse component in the cohort (69.8%; 35,614,081 mapped reads), and HPAXF049 with one of the lowest mouse components (7.5%; 3,109,853 mapped reads). Despite the difference in coverage, high mouse gene expression correlation was achieved between these samples (*r* = 0.93; Figure S5), suggesting coverage was not a major confounding factor. Furthermore, no significant association was observed between the mouse clusters and mouse gender (*p* = 0.10) or tumor stage (primary or metastatic; *p* = 0.55), and only some association with mouse strain (*p* = 3.09E–04). This was possibly driven by membership of all athymic nude mice in clusters 1 and 4 (Figure 1E), otherwise, it was difficult to discern possible influence of mouse strain on dominant stromal cell type from these data alone.

### Inferring tumor-stroma crosstalk from inter-species gene expression correlations

Given the potential for heterogeneous recruitment of mouse stroma to the human tumor, we next performed a comprehensive expression correlation analysis between human and mouse components to generate hypotheses on tumor-stroma crosstalk. We initially looked for evidence of a known tumor-stroma association, selecting the recent observation that fibroblast activation is associated with a tumor epithelial cell type [13]. Expression of human epithelial cadherin (*CDH1*) and epithelial cell adhesion molecule (*EPCAM*) were used as epithelial markers, and mouse expression of fibroblast associated protein (*Fap*), chondroitin sulfate proteoglycan 4 (*Cspg4*) and alpha-smooth muscle actin (*Acta2*) as CAF markers. In five of the six possible cross-species comparisons, a significant Pearson correlation coefficient (*p* < 0.05) was observed between the human epithelial and mouse CAF markers (Table 1; Figure S6A–S6F), particularly between *EPCAM* and *Fap* (*r* = 0.37, *p* = 7.90E–4; Figure S6A) indicating the positive relationship between tumor epithelial cell type and fibroblast activation exists in the PDX panel. No significant correlation was observed between the mouse CAF markers and two human mesenchymal cell type markers vimentin (*VIM*) and zinc finger E-box binding homeobox 1 (*ZEB1*; Table 1), with *Fap* achieving weak anti-correlation with both *VIM* (*r* = −0.20, *p* = 0.08) and *ZEB1* (*r* = −0.21, *p* = 0.06). Within the context of all correlations between human *CDH1* and 2495 most highly expressed and variable mouse genes, both *Fap* and *Cspg4* were ranked in the top 6% most correlated genes (Table S7). Notably, a significant number of these top 6% genes play a role in cell migration (*p* = 8.37E–13) and vasculature development (*p* = 3.12E–10), both characteristic of an activated stroma, including the top ranked gene collagen, type VIII, alpha 1 (*Col8a1*, *r* = 0.67; Figure S7), a key component of blood vessel endothelia.

**Table 1:** Pearson correlation coefficients calculated between human epithelial and mouse CAF markers

Human genes	Mouse genes		
	*Acta2*	*Fap*	*Cspg4*
*CDH1*	0.29**	0.24*	0.32**
*EPCAM*	0.06	0.37**	0.27*
*VIM*	0.01	−0.20	0.10
*ZEB1*	−0.01	−0.21	−0.05
**p < 0.01, *p < 0.05			

**Figure 2:**
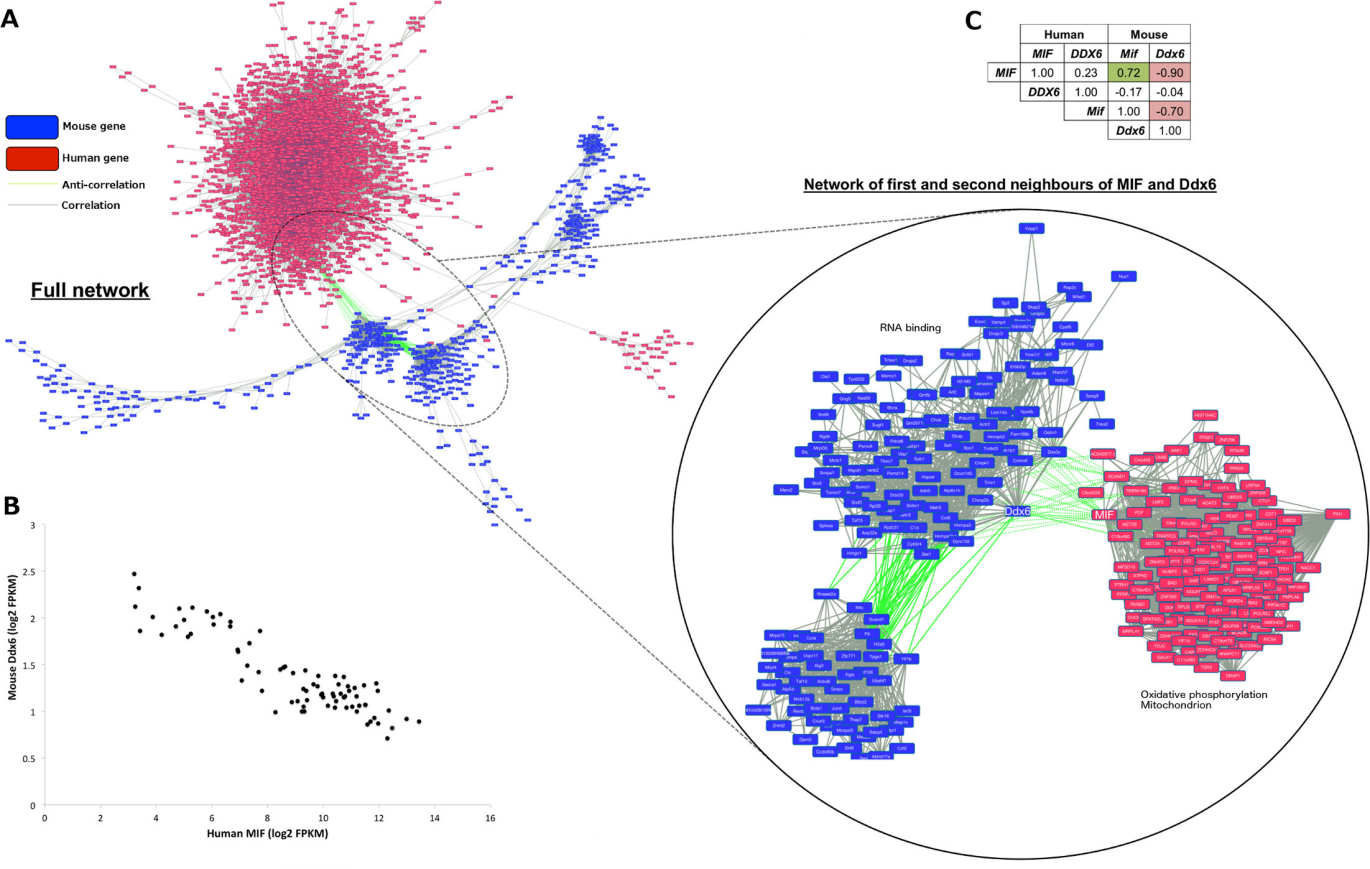
Human *MIF* and mouse *Ddx6* are strongly anti-correlated and are identified as cross-species hubs (**A**) Cytoscape [59] rendered human (red boxes) and mouse (blue) gene co-expression network where nodes are genes and edges indicate gene pairs achieving *r* > 0.85 (grey) or *r* < −0.85 (green). Magnified view shows sub-network of first and second neighbors of human *MIF* and mouse *Ddx6*. (**B**) scatterplot showing anti-correlation (*r* = −0.90) between human *MIF* and mouse *Ddx6*. (**C**) *r*-values between all combinations of human/mouse *MIF* and *DDX6* mRNA expression profiles.

To explore potentially novel gene-wise associations between human tumor and mouse stroma, an all-against-all comparison of 14,336 expression profiles representing the most highly expressed and variable genes from both the human (11,841 genes) and mouse (2495) components was performed. The resulting co-expression network consisted of 259,627 edges and 7089 nodes (Figure 2A) where nodes represented genes and edges were drawn between gene pairs achieving a stringent threshold of |*r*| > 0.85 (*p* < 2.20E–16) to control for false positives. The majority of edges (256,922) were between positively correlated human gene pairs, with the remainder of the network comprising of 2661 edges between mouse gene pairs, and 44 cross-species anti-correlated gene pairs. The strongest cross-species anti-correlation (*r* = −0.90) was observed between human macrophage migration inhibitory factor (*MIF*) and mouse DEAD-box RNA helicase 6 (*Ddx6*) (Figure 2B). In addition, human *MIF* (connected to 17 mouse genes) and mouse *Ddx6* (connected to 8 human genes) represented the most connected cross-species nodes in the network (Table S8).

Increased *MIF* expression and reduced *DDX6* expression are both known drivers of angiogenesis and strongly associated with VEGF activity, as such they have been implicated in tumor response to hypoxia [14–15]. Consistent with their known roles, positively correlated first or second neighbors of *MIF* in the network (Figure 2A) were enriched for mitochondrial processes (*p* = 7.15E–05) and oxidative phosphorylation (*p* = 1.72E–04), whereas mouse genes neighboring *Ddx6* were enriched for other RNA binding genes (*p* = 3.93E–08). To test the association with hypoxia more explicitly, models were divided into *MIF* high/*Ddx6* low (log_2_ FPKM human *MIF* > 11.5, mouse *Ddx6* < 1.05) and MIF low/Ddx6 high (*MIF* < 7.0, *Ddx6* > 1.8) corresponding to possible hypoxic and normoxic samples respectively (Table S9). Genes differentially expressed (log_2_ fold change (FC) > 1.50, false discovery rate (FDR) < 0.05) between the two groups were then identified. According to Toppgene, genes over-expressed in *MIF* high/*Ddx6* low samples were enriched (*p* = 1.50E–06) for signatures representing genes up-regulated under hypoxia *in vivo* [16]. Conversely, genes over-expressed in *MIF* low/*Ddx6* high samples were enriched (*p* = 5.09E–05) for genes down-regulated in the same study, thus supporting the association of high human *MIF* and low mouse *Ddx6* expression with tumor hypoxia.

Closer inspection of all possible inter- and intra-species correlations between *MIF* and *DDX6* (Figure 2C) revealed no significant association (*p* < 0.01) between human *DDX6* and either human *MIF* (*r* = 0.23, *p* = 0.04), mouse *Ddx6* (*r* = −0.04, *p* = 0.75), or mouse *Mif* (*r* = −0.17, *p* = 0.14). By contrast, human *MIF* and mouse *Mif* achieved the second highest correlation (*r* = 0.72) between human and mouse orthologs, suggesting that *MIF* has complementary roles in the tumor and mouse stroma.

### PDX models as a source of tumor and stroma specific markers of disease subtype

We next focused on lung and breast cancer as the two most highly represented diseases in the PDX cohort, in order to assess the potential of our approach to identify clinically relevant, independent human tumor and mouse stroma markers of disease subtype.

### Stromal-specific markers of lung and breast cancer subtype

**Figure 3:**
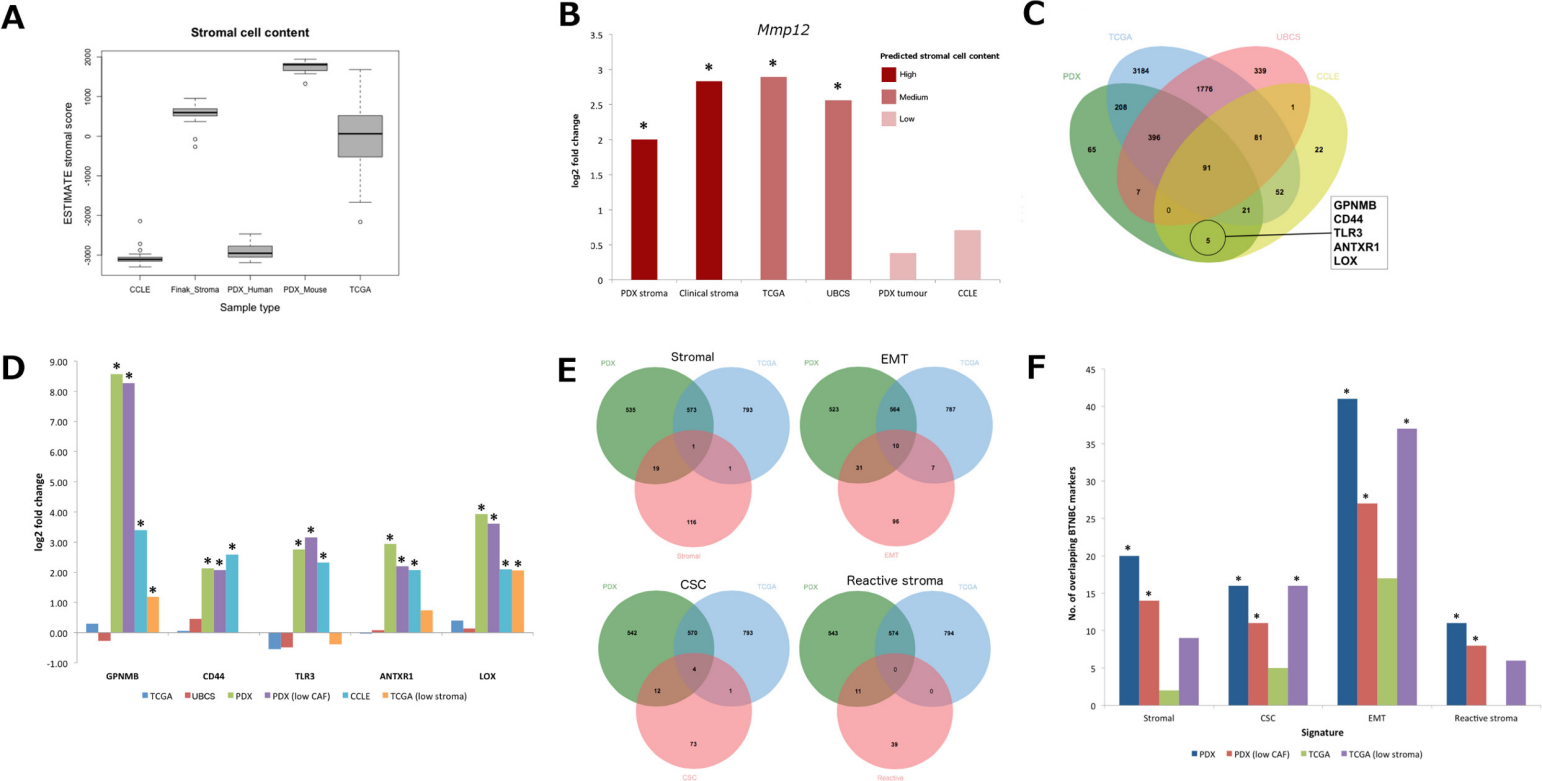
Compartment-specific gene expression markers of BTNBC (**A**) comparison of ESTIMATE stromal score between PDX, TCGA, CCLE and clinical stroma samples. (**B**) fold changes achieved by *MMP12* between BTNBC and ER+ luminal-B samples across all platforms (*p < 0.01). (**C**) derivation of tumor specific BTNBC markers exclusive to cell line and PDX datasets. (**D**) emergence of tumor specific markers in low stroma TCGA samples (*p < 0.05). (**E**) overlap of stromal [10], EMT [20], CSC [21] and reactive stroma [22] signatures with BTNBC markers derived from human PDX and TCGA samples. (**F**) comparison of log_10_ *p*-values achieved across sample types by overlap with each of the four signatures (*p < 0.05). CSC: cancer stem cell, EMT: epithelial-mesenchymal transition, *MMP12*: Matrix Metalloproteinase 12.

Comparison between mouse components of 14 lung adenocarcinomas and 18 squamous carcinomas identified no significantly differentially expressed genes, suggesting uniform recruitment of mouse stroma to human lung xenografts. By contrast, in the breast cancer panel, 18 mouse genes achieved significant fold change (|log2 FC| > 1.5, FDR < 0.05) between four ER+ luminal-B and nine basal-like TNBCs (BTNBC; Table S10). Such a low number of genes is in broad agreement with a dataset of patient tumor stroma samples isolated by laser capture micro-dissection (Finak_Stroma) [17] in which only 29 genes were differentially expressed (|log2 FC| > 1.5, FDR < 0.05; Table S12). However, there was little overlap between the two gene lists with matrix metallopeptidase 12 *(Mmp12)* the only gene over-expressed in both mouse (log_2_FC = 2.00, FDR = 2.43E–02) and clinical (log_2_FC = 2.83, FDR = 3.14E–09) stroma. Interestingly, whilst *MMP12* expression was largely absent in the PDX human component (mean log_2_ FPKM = 0.44) and cell lines of the Cancer Cell Line Encyclopaedia [18] (CCLE; mean log_2_ signal = 4.51) representing pure tumor cell populations (Figure 3A), significant over-expression in BTNBC was detected in clinical samples from The Cancer Genome Atlas [19] (TCGA; log_2_FC = 2.89, FDR = 2.67E–14) and the Utah Breast Cancer Study (UBCS; log_2_FC = 2.56, FDR = 1.21E–02; Figure 3B). Thus significant fold changes appear restricted to only those samples containing recruited stroma (PDX mouse component, Finak_Stroma, and clinical tumor samples), suggesting that *MMP12* expression is specific to BTNBC stroma, and absent in tumor cells.

### Tumor-specific markers of BTNBC

Exploiting the tumor purity of the PDX human component, we derived a putative set of tumor-specific BTNBC markers by comparing a list of 1127 genes over-expressed (log_2_FC > 1.5, FDR < 0.05) in BTNBC from the PDX human component with 273 genes over-expressed (log_2_FC > 1.5, *p* < 0.001) in cell lines. This resulted in an overlap of 117 genes on platforms representing high tumor content (Figure 3A). We then performed a second overlap between this tumor-specific gene signature and 6156 differentially expressed genes detected in TCGA and UBCS at FDR < 0.05 but ignoring fold change magnitude. By doing so, we reasoned that genes unique to the intersection between PDX and cell line datasets represented BTNBC markers that could only be observed in samples with high tumor purity, and overlooked in clinical samples containing a mixture of tumor cells and recruited stroma. Five genes fulfilled the criteria (Figure 3C): transmembrane glycoprotein NMB (*GPNMB*), *CD44*, toll-like receptor 3 (*TLR3*), anthrax toxin receptor 1 (*ANTXR1*) and lysyl oxidase (*LOX*). All five genes retained significant differential expression after removal of five breast cancer samples (HBCX10, HBCX11, HBCX24, HBCX6 and HBCX9) expressing CAF markers *FAP*/*CSPG4* in the human component, addressing the possibility that persistent retention of patient stroma in the BTNBC PDX models was confounding results.

To reproduce the tumor purity of the PDX human component and cell lines in clinical samples, we performed a second comparison between 12 BTNBC and 10 ER+ luminal-B TCGA patients representing samples with the highest estimated tumor cell content (ESTIMATE stromal score < −700). Remarkably, significant differential expression emerged in *GPNMB* (log_2_FC = 1.19, FDR = 1.18E–02) and *LOX* (log_2_FC = 2.07, FDR = 2.53E–05) with the fold change of a third gene, *ANTXR1* also increasing but not to significance (log_2_FC = 0.74, FDR = 0.13; Figure 3D). This suggests that at least two of the five genes are BTNBC markers expressed in patient samples, whose significance is masked by the difficulty of separating recruited stroma from tumor cells of stromal phenotype.

### Tumor-specific BTNBC markers are enriched for genes associated with a stromal phenotype

To gain further insight into potential BTNBC markers derived from high tumor content samples, we next compared 1127 and 1368 genes over-expressed (log_2_FC > 1.5, FDR < 0.05) in BTNBC from the PDX human component and TCGA patient samples respectively. This resulted in an overlap of 574 genes.

We initially speculated that the differences between the lists could in part be attributed to the presence of recruited stroma in typical clinical samples that is absent in the PDX human component (Figure 3A), so we included a 137-stromal gene signature [10] (Table S13A) in the comparison. Surprisingly, we observed greater overlap (*p* = 6.00E–04 by hyper-geometric test) of stromal signature genes with BTNBC markers derived from the PDX human component than those from clinical samples (*p* = 1.00; Table 2; Figure 3E; Table S14). To understand whether this overlap was indicative of specific cell type, we also overlaid epithelial-mesenchymal transition [20] (EMT; Table S13B), breast cancer stem cell [21] (CSC; Table S13C) and reactive stromal [22] (Table S13D) signatures. Clear enrichment (*p* = 7.77E–16) of mesenchymal markers including *VIM* was observed in the BTNBC PDX human component. By contrast, only a slight enrichment was observed (*p* = 0.07) in clinical BTNBC samples and whilst *VIM* achieved significant fold change, it was small (log_2_FC = 1.13, FDR = 2.15E–15) compared to that observed from the PDX human component (log_2_FC = 4.49, FDR = 2.18E–05). There was no enrichment of stromal or mesenchymal cell markers in genes over-expressed across either PDX or clincal ER+ luminal-B samples (Table 2; Table S14). The BTNBC PDX human component was also enriched for reactive stroma (*p* = 6.08E–04) and breast CSC markers (*p* = 1.60E–04; Table 2; Table S14), and whilst a high number of non-CSC markers were also present, the enrichment was not significant (*p* = 0.53). Furthermore, CD44, a marker for cells with tumor initiating potential, was identified as a BTNBC marker in both PDX (log_2_FC = 2.14, FDR = 5.91E–04) and cell line (log_2_FC = 2.58, *p* = 8.66E–03) but not in clinical (log_2_FC = 0.06, FDR = 0.79) datasets. Low CD24 expression is also indicative of tumor initiating potential, however human CD24 expression was not detected across any of the PDX samples. Conversely, non-CSC markers were enriched in genes over-expressed in PDX (*p* =1.26E–03) and TCGA ER+ luminal-B samples (*p* = 1.29E–05).

**Table 2:** Overlap of gene signatures with genes over-expressed in BTNBC and ER+ luminal-B breast cancers

Signature	BTNBC				ER+ luminal-B			
	PDX (1127^a^)	PDX FAP/CSPG4 low (793^a^)	TCGA (1368^a^)	TCGA low stroma (1763^a^)	PDX (511^a^)	PDX FAP/CSPG4 low (569^a^)	TCGA (876^a^)	TCGA low stroma (847^a^)
Stromal (137^b^)	20*	14*	2	9	1	5	4	6
CSC Up (90^c^)	16*	11*	5	16*	2	3	1	1
CSC Down (211^c^)	14	12	13	18	15*	14*	27*	29*
EMT Up (144^d^)	41*	27*	17	37*	5	11*	8	11
EM Down (156^d^)	30*	22*	34*	33*	8	6	21*	21*
Reactive stroma (50)	11*	8*	0	6	0	0	1	1

* *p* < 0.05 by hyper-geometric test.

a Number of genes over-expressed in TNBC or ER+ luminal-B.

b From [10].

c Breast cancer stem cell (CSC) signature from [21].

d Epithelial-mesenchymal transition (EMT) signature from [20].

e From [22].

Encouragingly, BTNBC markers remained enriched for stromal (*p* = 3.30E–03), EMT (*p* = 4.17E–10), CSC (*p* = 1.59E–03) and reactive stromal (*p* = 3.04E–03) signatures (Figure 3F; Table 2; Table S14) after removal of the five breast PDX models expressing CAF markers *FAP* and *CSPG4* suggesting the presence of stromal-like signature genes in the PDX human component was independent of patient stroma retention. Furthermore, there was no evidence of bias towards mesenchymal or mesenchymal stem-like (MSL) TNBC subtypes [23] (Table S11), indeed no MSL or immunomodulatory (IM) subtypes were present in the PDX data in accordance with the view that these subtypes are likely defined by high expression of genes from the micro-environment rather than the tumor itself [24]. Therefore our observations are unlikely due to over-representation of the stromal subtype in PDX BTNBC models.

### Stromal phenotype emerges in BTNBC clinical samples of high tumor content

As before, we reproduced the tumor purity of the PDX human component in clinical samples by focusing on TCGA samples with the lowest predicted stromal cell content (ESTIMATE stromal score < −700). Remarkably, enrichment of both EMT (*p* = 2.05E–07) and CSC (*p* = 1.83E–02) signatures emerged in genes differentially expressed between the remaining 12 BTNBC and 10 ER+ luminal B TCGA samples, comparable to that observed in the PDX human component (Figure 4C). The numbers of stromal and reactive stromal signature genes also increased although not sufficient to achieve significance. Taken together, these findings support the presence of a subpopulation of cells with CSC and mesenchymal features in BTNBC that is difficult to detect in a typical clinical sample due to equivalent expression signals from the recruited stroma.

## DISCUSSION

We describe the output of a comprehensive expression analysis of 79 pan-cancer PDX models using an RNA-Seq generated species-specific mapping strategy. Accurate separation of human and mouse components of the tumor allows use of PDX tumors to gain unique insights into tumor-stroma crosstalk, and through cross comparison, generate both tumor and stroma expression signatures that give insights specific to disease subtypes, and also aid development of biomarker signatures. When passaged *in vivo* much of the patient stroma present upon implantation and early passages is eventually replaced by murine stroma, and whilst this can lead to loss of some original features found in the patient tumor microenvironment [25], it is key to enabling non-invasive species-specific separation of PDX tumor from stroma *in silico*. Therefore, it offers a unique opportunity not only to look at features specific to the stroma but also reveal new biology within the human tumor cells.

### Identification of distinct mouse stromal clusters defined by dominant cell type

Mouse gene expression profile clustering revealed distinct PDX stromal subtypes broadly related to dominant cell types characterized by adipocyte, embryonic stem, and myeloid signatures, the latter indicating a high level of inflammatory infiltrate in the mouse stroma. These were independent of cancer type and PDX provider and suggest heterogeneous recruitment of mouse stroma to the human tumor. Since interaction between the tumor and stroma is known to influence drug response, our findings imply that the effect of the dominant stromal cell type in an individual model should be considered when analyzing therapeutic response. This is particularly pertinent for compounds that may drive efficacy through stromal elements, or be subject to stromal derived resistance. However, whilst the approach provides the opportunity to explore the association of stromal subtype and the degree of inflammatory infiltrate with survival outcome and drug resistance, it is important to characterize the models carefully. In this study of commonly used models there was a notable enrichment of high-grade tumors with poor prognosis [26], which will influence the outcome of such studies.

### Human-mouse gene correlation analysis identifies both known and potentially novel tumor-stroma associations

The degree of transcriptional heterogeneity in the mouse component, and the potential for active involvement of the xenograft in the process prompted us to look more closely at gene expression correlations between the human and mouse component. This approach identified known associations between tumor epithelial cell type and fibroblast activation [13], exemplified by significant correlation between human epithelial and mouse CAF markers. These were amongst several mouse genes correlated with human *CDH1* including numerous endothelial-associated genes. We then applied more stringent criteria to generate a cross-species correlation network and identify novel associations. The strongest relationship was an anti-correlation between human *MIF* and mouse *Ddx6*. *MIF* plays an important role in the induction of vascular endothelial growth factor (VEGF) activity promoting blood vessel growth [27], and is also a target of the transcription factor hypoxia-inducible factor 1-alpha (HIF1α) [14]. Conversely, *DDX6* inhibits VEGF protein expression under normoxia through binding to the 5′-UTR of its mRNA [15]. Our data suggest a potential stromal role for *DDX6* in the hypoxia response as an alternative to its previously established tumor-specific activity, and a potential link with *MIF* activity in the tumor.

Overall, our findings highlight the value of RNA-Seq as a sensitive approach to dissect the complex interplay between both species compartments. However there are two caveats. Firstly the original tumor-stroma interactions in the patient are unlikely to be completely recapitulated due to incompatibility of human and mouse proteins, and the use of immune-compromised mice to promote xenograft establishment. Whether this is major caveat remains to be established. Whilst our results suggest known interactions develop in PDX models, further work will be necessary to establish a direct functional link in any novel interaction suggested by these data alone, and whether it represents a clinically relevant association or one that is unique to the xenograft. Secondly, the majority of tumors were implanted subcutaneously providing an ectopic environment. Where technically achievable, comparison with a more physiological implantation site used in orthotopic engraftment may better recapitulate the original patient stroma, and as such models become more widely available, similar profiling studies should be performed on these.

### Stromal gene expression markers of cancer-subtype

Compared to the transcriptional differences observed between lung and breast cancer subtypes within the human tumor component, typically involving >1000 genes, the differences between the mouse stroma were small. Indeed, no mouse genes were deemed differentially expressed between lung adenocarcinoma and squamous mouse stroma, and only 18 mouse genes between breast ER+ and TN. This suggests recruitment of mouse stroma to the tumor is largely independent of disease subtype.

The search for stromal specific markers of BTNBC also highlighted potential disparities between PDX mouse and patient stroma. Whilst there was consensus in terms of fewer changes in stroma compared to tumor, only *MMP12*, previously associated with breast cancer poor prognosis [17] [28], was detected as a BTNBC marker in both PDX mouse and patient stroma. The patient stroma clearly reflected changes typically seen in the tumor between TNBC and ER+ samples in agreement with recent studies suggesting that intrinsic breast cancer subtypes can be recapitulated based on the transcriptome of cancer adjacent tissue [29–31]. By contrast, there was no evidence of subtype reflected in the PDX stroma transcriptome but instead genes differentially expressed in PDX stroma were enriched in functions characteristic of metastasis, a process common to BTNBC.

Whilst these results could reflect intrinsic differences between the patient and mouse stroma, alternatively they could be a consequence of the higher predicted stromal cell purity achieved with our RNA-Seq approach compared to laser capture micro-dissection. Therefore, the specificity achieved by RNA-Seq in disambiguating human from mouse could offer a significant advantage, particularly in combination with a systematic integration of clinical datasets such as TCGA to overcome limitations of using mouse stroma. For example, evidence for *MMP12* as a BTNBC stromal marker was strengthened by the observation that its expression is absent in samples containing only tumor cells, but present in samples that typically contain a significant stromal cell component.

### Expression signals from recruited stroma could mask presence of poor prognosis markers in BTNBC

A major outcome of the disease-specific analysis was the identification of a BTNBC stromal signature in the PDX human component, not detected in TCGA or UBCS clinical samples. This was achieved through the ability of RNA-Seq to differentiate the two major sources of cells with a stromal phenotype in a typical xenograft sample, trans-differentiated tumor cells [32] (human cells) and co-opted/recruited stroma (mouse cells). In a patient sample, this is difficult to accomplish with high confidence, even with specialist techniques such as laser capture micro-dissection, highlighting the capacity of RNA-Seq to refine complex signatures that are typically derived from heterogeneous cell populations.

The enrichment of breast CSC markers, including *CD44*, and EMT markers such as *VIM* supports previous work that showed EMT and CSC phenotypes are more likely to occur in basal breast cancers [33–34]. Notably, *CD44* was one of five genes previously associated with BTNBC and poor prognosis that were clearly over-expressed in BTNBC PDX human and cell line samples but not in clinical samples. Of the other four genes, *TLR3* and *ANTXR1* are also CSC markers [35–36], whilst *LOX* and *GPNMB* are metastatic markers of BTNBC [37–40]. Given the phenotypes associated with these genes, we could have uncovered a particularly aggressive sub-population of cells analogous to CSCs residual after conventional treatments [21] that are present in BTNBC but absent in ER+ luminal-B patients. Critically, the observation that CSC and EMT gene signatures emerge in BTNBC markers derived from a dataset restricted to TCGA samples with the lowest stromal cell content strongly suggests that the phenomenon is not exclusive to PDX models, and counters the possibility that CSCs are simply more successful at engrafting in the mouse mammary microenvironment than other cell types [41].

We had two further concerns about our PDX breast cancer dataset, firstly BTNBC is over-represented due to the difficulty in establishing ER+ tumors in mice [42], and secondly, despite the retention of poor prognosis markers after removal of five BTNBC PDX models expressing CAF markers, we could not completely rule out the possibility of persistent patient stroma retention confounding the above observations. If this is the case, the presence of stromal-associated genes in the BTNBC PDX human component could reflect the level of reactive stroma in the original patient tumor. This indicates increased metastatic capacity leading to disease progression and poor prognosis, particularly in BTNBC [43]. Recently, a study has shown that increased stroma has the opposite effect in ER+ breast tumors [44], and although the results have since been challenged [45], a bias towards aggressive tumors with poor prognosis in PDX models could lead to an enrichment of high stroma BTNBC and low stroma ER+ luminal-B tumors in our breast cancer cohort and the appearance of stromal-associated genes not present in the clinical data. Indeed, ER+ luminal-B tumors are themselves distinct from other ER+ subtypes due to their aggressive phenotype and resistance to conventional hormone therapy [46]. Therefore, the effect of excluding mouse stroma is to reveal genes specific to the original reactive stroma in the patient.

One further possibility was we had simply identified the presence of a stromal-like BTNBC subtype, however we found no evidence of bias towards mesenchymal or MSL TNBC subtypes [23] in our breast xenografts, nor in our TCGA/UBCS clinical samples of high tumor purity in which the signature emerged.

Despite these concerns, the masking of key markers of BTNBC by expression signals from recruited stroma is a significant observation and clearly illustrates the confounding effects of tumor sample heterogeneity on differential expression measurements. Considering the importance of the genes missed in the clinical samples unless stromal recruitment is accounted for, our observations have implications for any study that uses clinical samples to derive cancer-associated signatures, and potentially contribute to the poor overlap observed between signatures derived from different studies. They also demonstrate the potential of the RNA-Seq approach to refine complex signatures whose source compartment is ambiguous.

## CONCLUSIONS

This study has established RNA-Seq as a cost-effective approach to enable simultaneous analysis of human tumor and mouse stroma across a pan-tumor explant panel. The analytical strategy provided new insights into mouse stromal heterogeneity and compartment-specific disease markers otherwise overlooked by other technologies. Whilst further work is necessary to investigate the clinical relevance of the findings, they highlight the use of the technology as a platform to explore mouse stroma recruitment to the human tumor, and, critically, how this may influence therapeutic response. The strategy can also be applied routinely in pharmacodynamic studies of PDX models for detailed monitoring of compartment specific changes after treatment, ultimately leading to better efficacy prediction in the patient. As a resource for the pre-clinical model research community, all gene-level human and mouse expression data have been deposited in the ArrayExpress database (www.ebi.ac.uk/arrayexpress) under accession number E-MTAB-3980.

## METHODS

### Ethics statement

All animal studies were conducted in accordance with U.K. Home Office legislation, the Animal Scientific Procedures Act 1986, as well as the AstraZeneca Global Bioethics policy. All experimental work is outlined in project license 40/3483, which has gone through the AstraZeneca Ethical Review Process. Studies in the United States were conducted in accordance with the guidelines established by the internal IACUC (Institutional Animal Care and Use Committee) and reported following the ARRIVE (Animal Research: Reporting *in vivo* experiments) guidelines.

### Animals

Female C.B.-17 severe combined immunodeficient (SCID) mice were purchased from Charles River Laboratories (Wilmington, MA). NOD/SCID/IL2Rγnull (NSG) mice were purchased from Jackson Laboratories (Bar Harbor, ME). Beige Nude XID mice were purchased from Harlan Laboratories (Madison, WI). Mice were housed under pathogen-free conditions in individual ventilated cages (IVC) at our Association for the Assessment and Accreditation of Laboratory Animal Care (AAALAC) accredited facility in Waltham, MA. All animal manipulations were conducted in a biosafety cabinet maintained under positive pressure.

### PDX model establishment

In-house PDX models were established from fresh patient tissue procured from Maine Medical Center BioBank (Portland, ME) and consented according to the Human Biological Samples Policy. Samples were received within 24 hours of surgery, minced and implanted subcutaneously into either NSG or CB17 SCID mice. The HBXF-079 sample was implanted orthotopically. The CTC-174 model was developed from circulating tumor cells in patient blood samples procured from Conversant Biologics (Huntsville, AL) and consented according to the Human Biological Samples Policy. The cells were isolated and implanted into the mammary fat pad of NSG mice. In addition, several models were purchased from Jackson Laboratories (Sacramento, CA) as tumor-bearing NSG mice. In most cases, the models were serially transplanted into fresh NSG mice as fragments for expansion and characterization, except BR0869F. For the remaining models, small pieces of PDX derived tumor were purchased specifically for profiling from Experimental Pharmacology and Oncology (EPO; http://www.epo-berlin.com), Xentech (http://www.xentech.eu) and Champions (http://championsoncology.com) representing athymic nude, SCID, or NSG mice (Table S1).

### RNA extraction

∼50 mg of tissue were cut from the frozen tumors and RNA isolated using the Qiazol kit with a DNase digestion using the RNase-free DNase Kit (Qiagen) on the Qiacube according to manufacturer's instructions. RNA concentration was measured using the NanoDrop ND1000 (NanoDrop), and quality determined using the Agilent RNA nano 6000 kit and Bioanalyzer (Agilent Technologies). RNA integrity numbers (RIN) for all samples fell between 7 and 10.

### RNA-Seq data

RNA libraries were made with the Illumina TruSeq RNA Sample Preparation kit (un-stranded) according to the manufacturer's protocol. These libraries were then submitted for 100 bp paired-end sequencing on the Illumina HiSeq 2000 platform using one lane per three to six PDX models. A concatenated human (GRCh37/hg19) and mouse (GRCm38/mm9) genome was then constructed to form a single genome of 43 chromosomes (23 from human and 20 from mouse). This was indexed using StarAlign [48] and a “gtf” formatted file combining annotations from both human and mouse genes downloaded from Ensembl version 75 [47]. The length of donor/acceptor sequence (“sjdbOverhang”) either side of a splice junction was set to 99 bases, with all other parameters set to their defaults. The sequenced reads were aligned to the human-mouse genome using StarAlign [48] with no more than three mismatches across each end of the pair allowed, and reads mapped to multiple locations discarded. Reads whose ratio of mismatches to mapped length was greater than 0.10, and non-canonical splice junctions were also removed. All other parameters were set to their defaults for non-stranded alignment. Since we only considered reads uniquely mapped to the human-mouse genome according to the mapping parameters above, reads mapping to both human and mouse genomes were automatically discarded. Therefore, the output of the pipeline was a set of species-specific reads that mapped uniquely either to the human or mouse genome.

### Measurement of expression level

The number of reads overlapping each gene present in both human (GRCh37) and mouse (GRCm38) annotation files downloaded from Ensembl version 75 [47] were calculated using the R Bioconductor package HTSeq [49] in un-stranded, union mode with all other parameters set to default values. For the same group of genes, expression based on Fragments Per Kilobase per Million fragments mapped (FPKM) was estimated using Cuffnorm with library type defined as “fr-unstranded” and all other parameters set to defaults [50]. Non-protein coding genes were ignored, as well as genes whose largest transcript is less that 400 bp due to potential over-estimation of expression across transcripts less than the average fragment length. For PDX model BR0555 represented by two biological and three technical replicates, the replicate whose mouse component achieved the highest ESTIMATE stromal score was chosen for downstream analyses. Where required, mouse genes names were converted to human and vice versa using MammalHom (http://depts.washington.edu/l2l/mammalhom.html).

### Clustering of gene expression data with consensus non-negative matrix factorization (NMF)

We applied NMF to cluster the human and mouse transcriptomes and identify tumor and stromal specific subtypes respectively. The underlying principle of NMF is dimensionality reduction in which a small number of meta-genes, each defined as a positive linear combination of the genes in the expression data, are identified and then used to group samples into clusters based on the gene expression pattern of the samples as positive linear combinations of these meta-genes. Using the R package *NMF* [51], factorization rank *k* was chosen by computing the clustering for *k* = 2–11 against 50 random initializations of both the actual and a permuted gene expression matrix, and selecting the *k* value achieving the largest difference between cophenetic correlation coefficients calculated from the actual and permutated data (Figure S8). For further visual confirmation of a sensible choice of *k*, consensus matrices were generated corresponding to different *k* values (Figure S9). To achieve stability, the NMF algorithm was then run against 200 perturbations of each gene expression matrix at the chosen values of *k* = 9 (human) and *k* = 5 (mouse).

### Breast cancer differential expression analyses

### PDX models

PDX model hormone receptor status was determined by immunohistochemistry, and broadly correlated with mRNA expression. Nine of the 10 TNBC PDX samples were basal-like and all four ER+ PDX samples luminal-B according to PAM50 classification [52] (Table S10) therefore comparisons were restricted to these two intrinsic subtypes. TNBC subtypes listed in Table S11A were predicted using TNBCtype [53] with FPKM expression values as input. Two tumors (HBCX7 and HBCX19) that had undergone metastasis were ignored. Differentially expressed genes were identified using the R Bioconductor package DESeq2 [54]. “minReplicatesForReplace” was set to seven or size of the smallest group if one group contained less than seven samples. All other parameters were set as defaults. Only genes achieving FPKM > 1 in at least one sample were input to DESeq2. Differential expressed genes were defined as those achieving a |log_2_FC| > 1.5 and FDR < 0.05.

### Patient stroma

Agilent-012391 Whole Human Genome Oligo Microarray G4112A expression data for a set of 45 human breast cancer samples whose stroma had been isolated from the tumor by laser micro-dissection [17] were downloaded from the Gene Expression Omnibus [55] (GSE9014). Raw expression data in the Agilent Feature Extraction text files were loess normalized and differential expression between seven TNBC and 38 ER+ samples calculated using the R Bioconductor package Limma [56]. Probesets achieving a |log_2_FC| > 1.5 and FDR < 0.05 were deemed differentially expressed.

### UBCS

Fresh frozen breast tissue samples were obtained from 88 women who had surgery at the Huntsman Cancer Hospital from 2009–2012, including tumor tissues from 69 breast cancer patients. One tumor sample yielded poor quality RNA (RIN = 2.5) and was removed from consideration, resulting in a panel of 68 tumor samples. RNA libraries were made with the Illumina TruSeq Stranded mRNA Sample Preparation kit with oligo dT selection according to the manufacturer's protocol. These libraries were then submitted for 50 bp single-end sequencing on the Illumina HiSeq 2000 platform using eight samples per lane. The resulting reads were aligned to the human (GRCh37/hg19) genome using StarAlign [48] with no more than three mismatches and only uniquely mapped reads allowed. Reads whose ratio of mismatches to mapped length was greater than 0.10 were also discarded. All other parameters were set to their defaults for stranded alignment. The number of reads overlapping each gene present in the human (GRCh37) annotation file downloaded from Ensembl version 75 [47] were calculated using the R Bioconductor package HTSeq [49] in stranded, union mode with all other parameters set to default values. The resulting gene-by-sample matrix consisted of 12 ER+ luminal B and 10 BTNBC samples. TNBC subtypes listed in Table S11B were predicted using TNBCtype [53] with FPKM expression values as input. Differentially expressed genes (|log_2_FC| > 1.5 and FDR < 0.05) were identified with DESeq2 [54] using the same protocol applied to the PDX models.

### TCGA

TCGA gene expression data (June 2014) processed using the RNASeqv2 pipeline were downloaded from the Broad Institute GDAC Firehose (http://gdac.broadinstitute.org) and parsed to generate a gene-by-sample matrix of raw counts across solid tumor samples only. Breast cancer samples treated with tamoxifen were discarded as were genes achieving a mean count < 1 across all remaining samples. The resulting matrix consisted of 18,488 genes across 79 luminal B ER+ and 54 TNBC samples as classified by TCGA Network [19] by PAM50 [52]. TNBC subtypes given in Table S11C were taken from [56]. Differential expression was calculated using DESeq2 [54] as for the PDX models.

### CCLE

RMA normalized Affymetrix U133+2 expression data was downloaded from CCLE [18], and all non-breast cancer cell lines were removed as well as cell lines for which subtype was ambiguous according to [58]. Since no PDX or TCGA basal-like samples were classified as claudin-low, these were removed from the cell line cohort. The resulting gene-by-sample matrix consisted of 12 ER+ luminal B and seven BTNBC samples. TNBC subtypes given in Table S11D were taken from [23]. Genes achieving |log2FC| > 1.5 and *T*-test *p*-value < 0.01 were defined as differentially expressed.

Note that for cross-platform comparisons, we considered only a core set of 15,984 genes represented on all platforms.

### Expression data preparation for clustering and cross-species correlation analysis

Gene-by-sample expression matrices were generated for each species in which only genes achieving FPKM > 10 (human genes) or FPKM > 2 (mouse genes) in at least one sample, and coefficient of variation > 0.20 were retained. The resulting matrix of FPKM expression values was converted to a non-negative matrix by addition of an arbitrary value of 1.1 to each entry and then taking log_2_ of the result.

### EMT and breast CSC signatures

The EMT signature was taken from [20] consisting of 144 human (141 mouse) genes up-regulated and 156 human (142 mouse) genes down-regulated in EMT according to [20]. The breast CSC signature was based on the CD44^+^/CD24^−/low^-mammosphere (MS) signature of [21] using 90 up-regulated and 211 down-regulated human genes.

### Mouse gender predictions

All genes on the Y chromosome were identified, and pseudogenes and genes achieving a count of < 1000 reads across all models were removed. The remaining genes were manually inspected, and those with evidence of expression across all models and therefore not following the profile of the majority of genes were also removed. Genes contributing to the mouse gender prediction consisted of *Eif2s3y*, *Ddx3y*, *Kdm5d* and *Uty*. Read counts across these genes were summed for all models and the results divided by the total read count across all genes and multiplied by 1000 to give a Y index. Mice achieving Y index > 10 were classified as male, and female otherwise.

## SUPPLEMENTARY MATERIALS FIGURES AND TABLES












