# Supplementary material for: Whole transcriptome profiling of patient-derived xenograft models as a tool to identify both tumor and stromal specific biomarkers
Source: Oncotarget. 2016 Mar 9;7(15):20773–87. doi: 10.18632/oncotarget.8014 (PMC4991491; doi:10.18632/oncotarget.8014)
Supplement: Supplementary file 1 [file oncotarget-07-20773-s001.pdf]

Whole transcriptome profiling of patient-derived xenograft models as a tool to identify both tumor and stromal specific biomarkers

Supplementary Materials

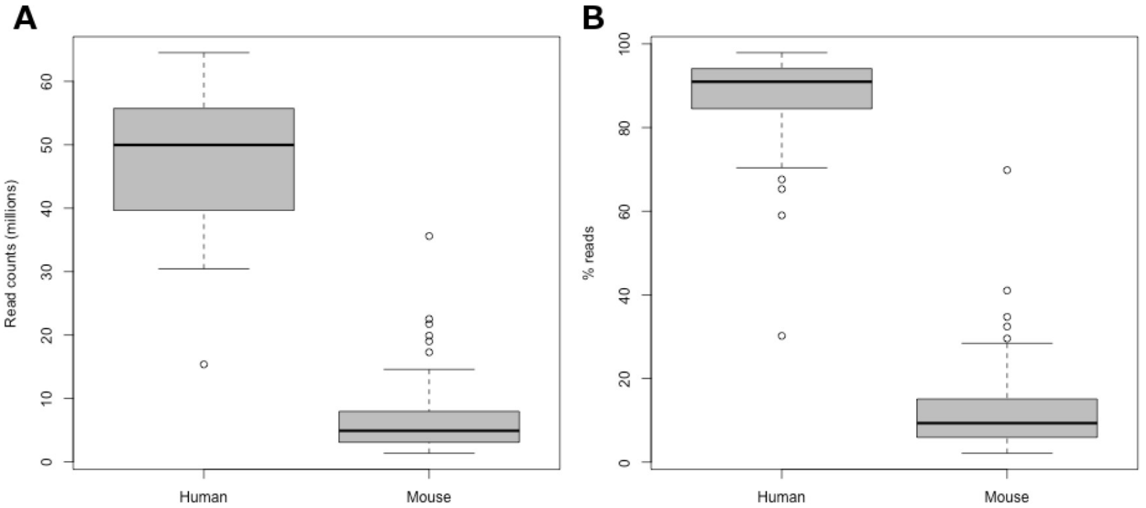

Supplemenatry Figure S1: Read mapping statistics. Boxplots showing (**A**) number and (**B**) percentage of reads mapping to human and mouse across the 79 PDX models.

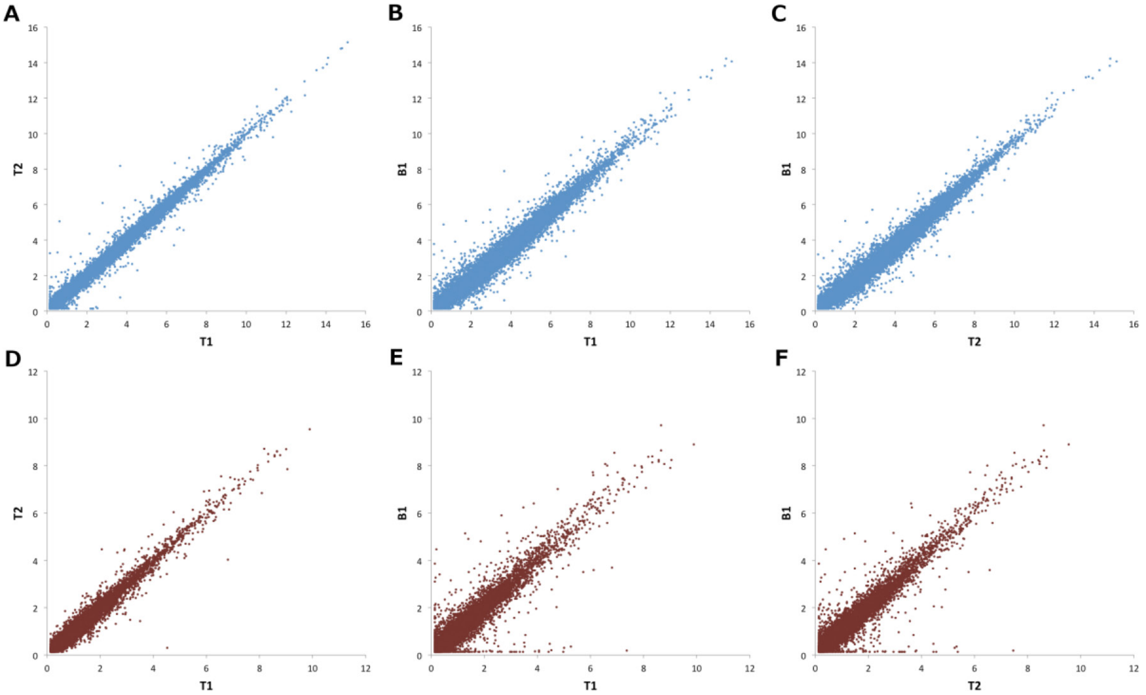

Supplemenatry Figure S2: Replicate comparison. Scatterplots showing expression correspondence between two technical and one biological replicate across human (represented by blue markers) and mouse (red) genes. (**A**) Technical replicate 2 (T2) versus 1 (T1) (human; $r = 0.99$), (**B**) biological replicate 1 (B1) versus T1 (human; $r = 0.99$), (**C**) B1 versus T2 (human; $r = 0.99$), (**D**) T2 versus T1 (mouse; $r = 0.99$), (**E**) B1 versus T1 (mouse; $r = 0.96$), (**F**) B1 versus T2 (mouse; $r = 0.96$).

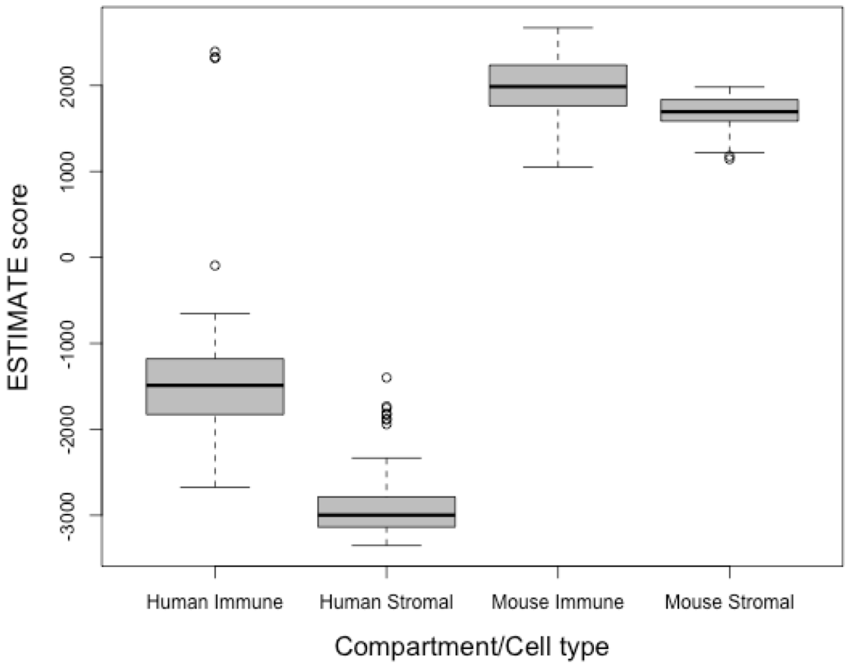

Supplemenatry Figure S3: Comparison of ESTIMATE [10] stromal and immune cell content in the human and mouse components.

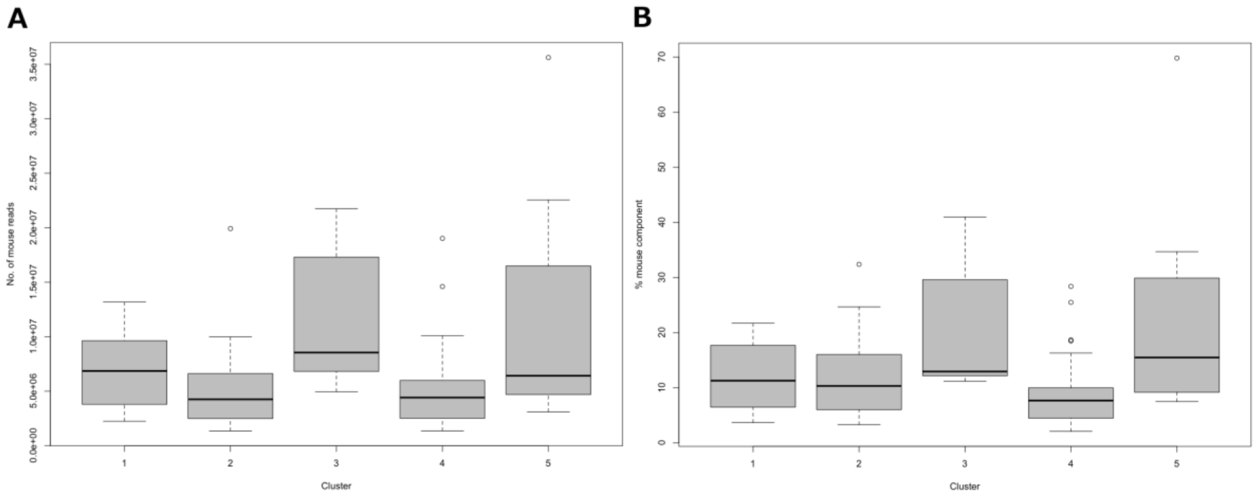

Supplemenatry Figure S4: Assessment of read coverage across mouse clusters. Comparison of (**A**) number of mapped mouse reads, and (**B**) percentage mouse component between mouse clusters.

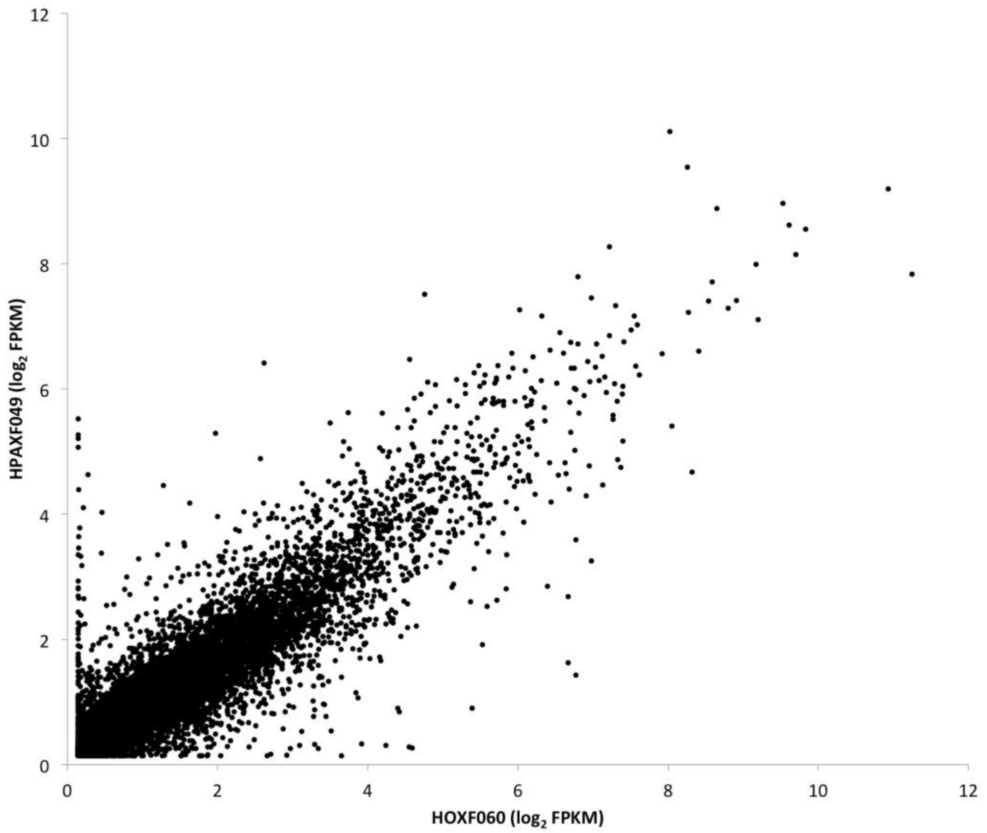

Supplemenatry Figure S5: Comparison of mouse gene expression between high and low coverage samples. Mouse gene expression correlation (*r* = 0.93) between HOXF060 (high percentage mouse component: 69.8%, high coverage: 35,614,081 reads mapped to mouse genome) and HPAXF049 (low percentage mouse component: 7.5%, low coverage: 3,109,853 reads).

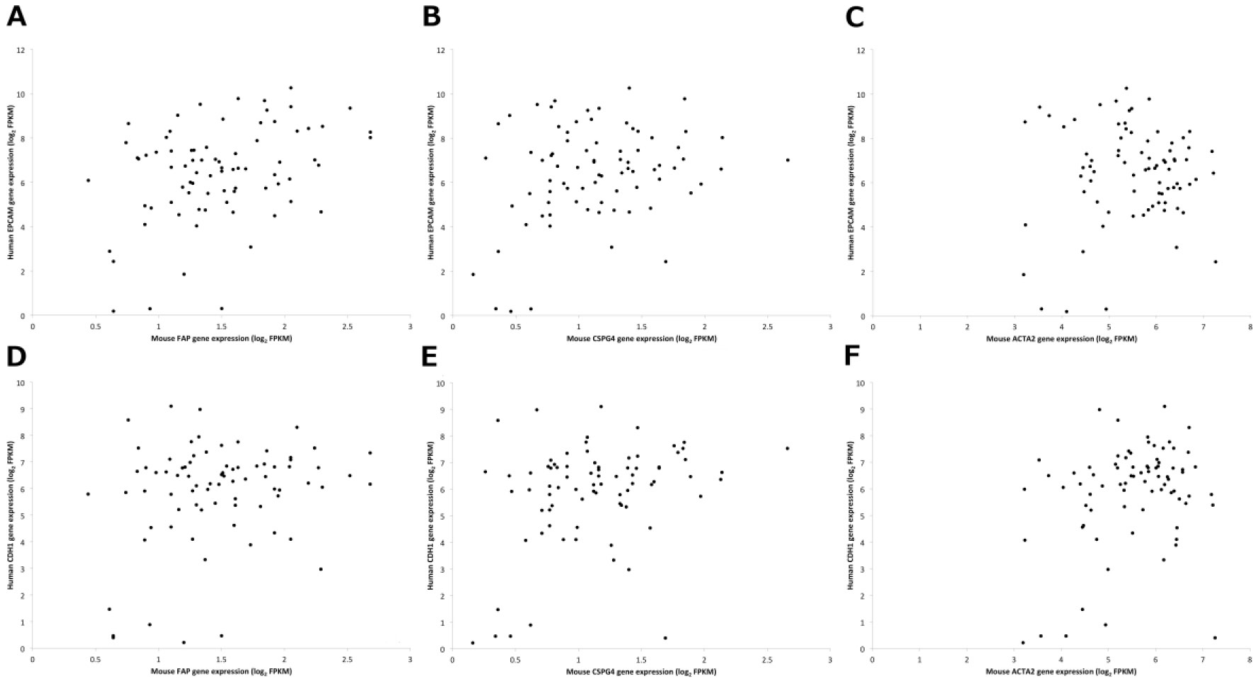

Supplemenatry Figure S6: Comparison of human epithelial and mouse cancer activated fibroblast markers. (**A**) *EPCAM* versus *Fap* (*r* = 0.37). (**B**) *EPCAM* versus *Cspg4* (*r* = 0.27). (**C**) *EPCAM* versus *Acta2* (*r* = 0.06). (**D**) *CDH1* versus *Fap* (*r* = 0.24). (**E**) *CDH1* versus *Cspg4* (*r* = 0.32). (**F**) *CDH1* versus *Acta2* (*r* = 0.29).

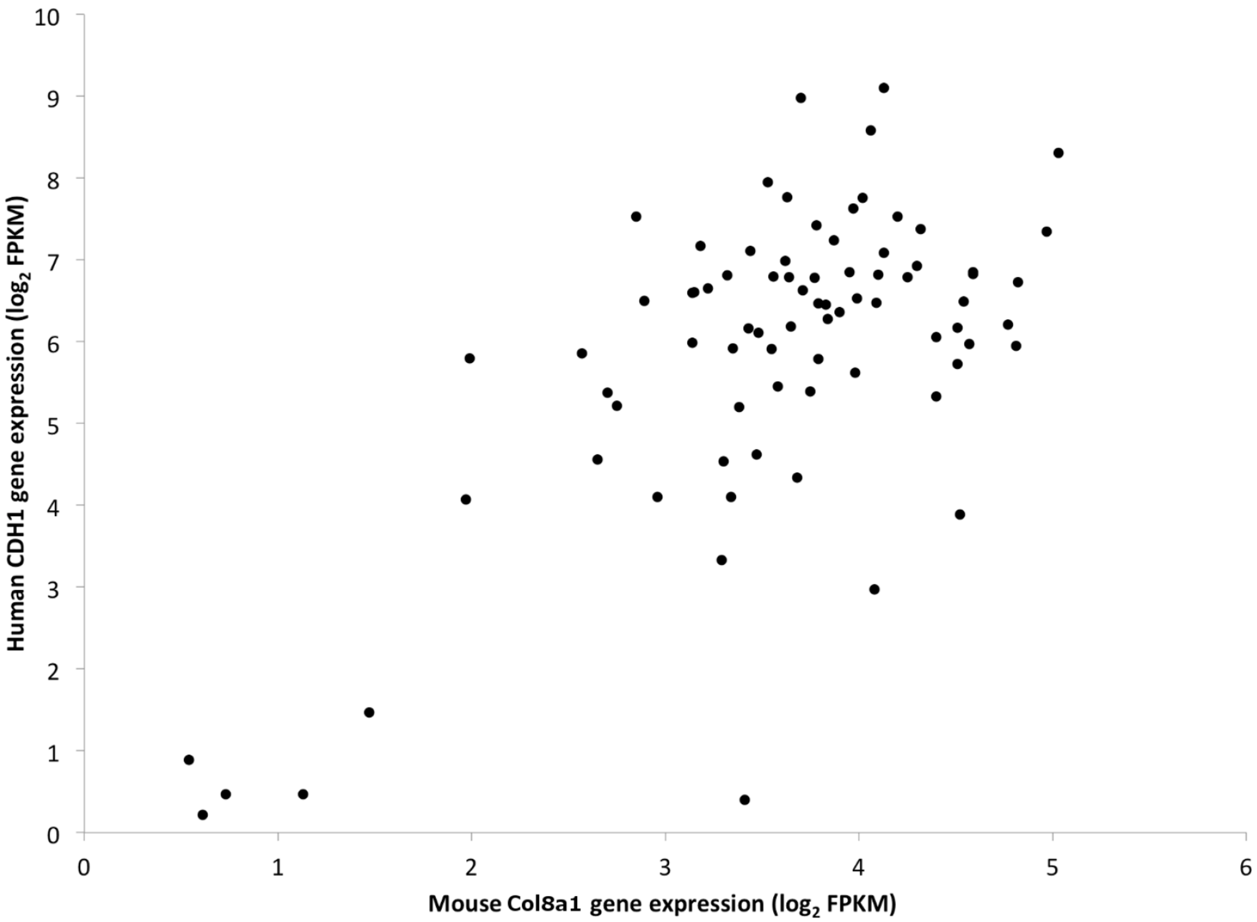

Supplemenatry Figure S7: Comparison between human *CDH1* and mouse *Col8a1* gene expression (r = 0.67).

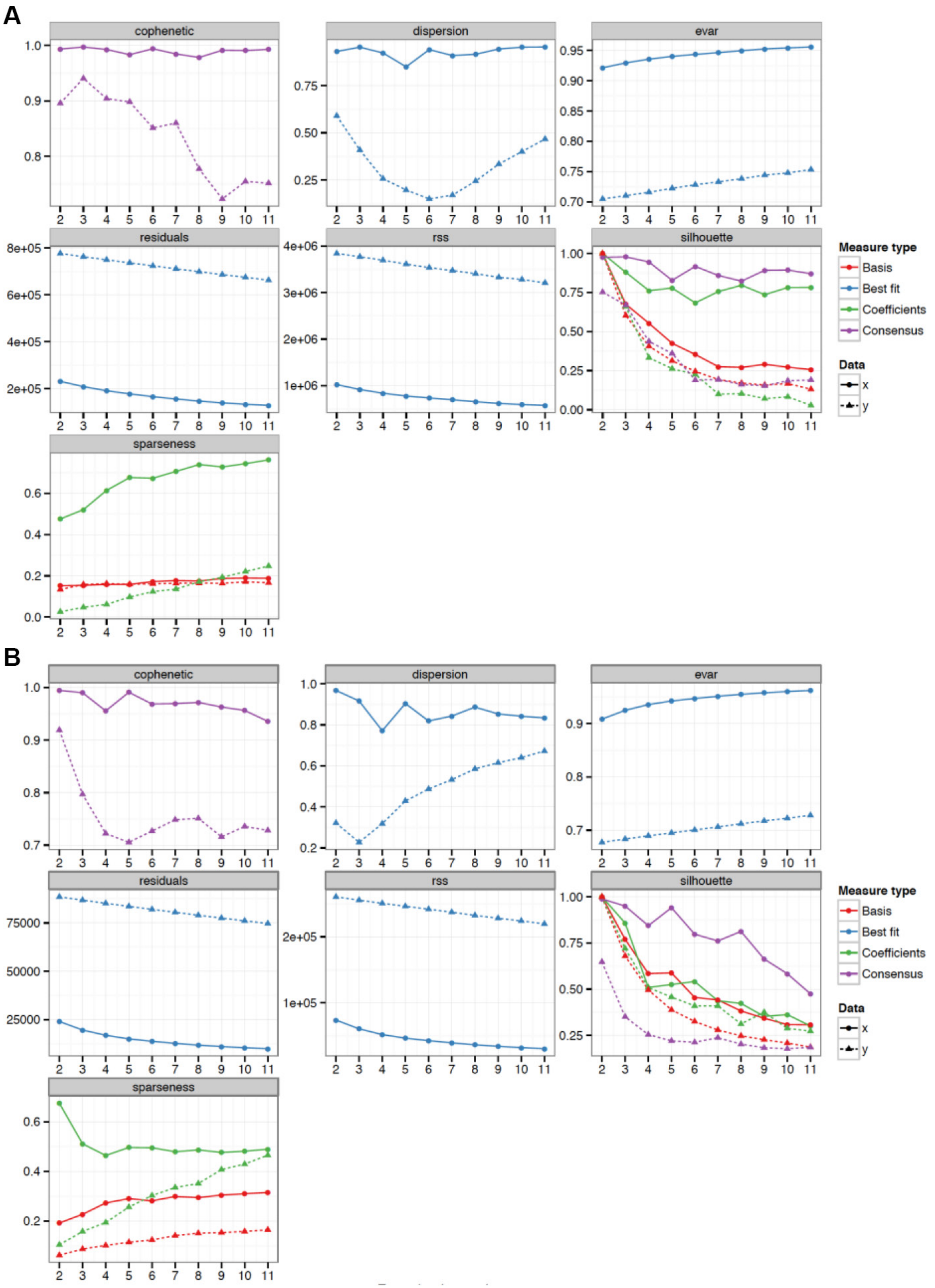

Supplemenatry Figure S8: Rank quality measures generated by the R package *NMF* [51]. Quality measures computed from 50 runs for each value of rank *k* across (**A**) human and (**B**) mouse expression datasets.

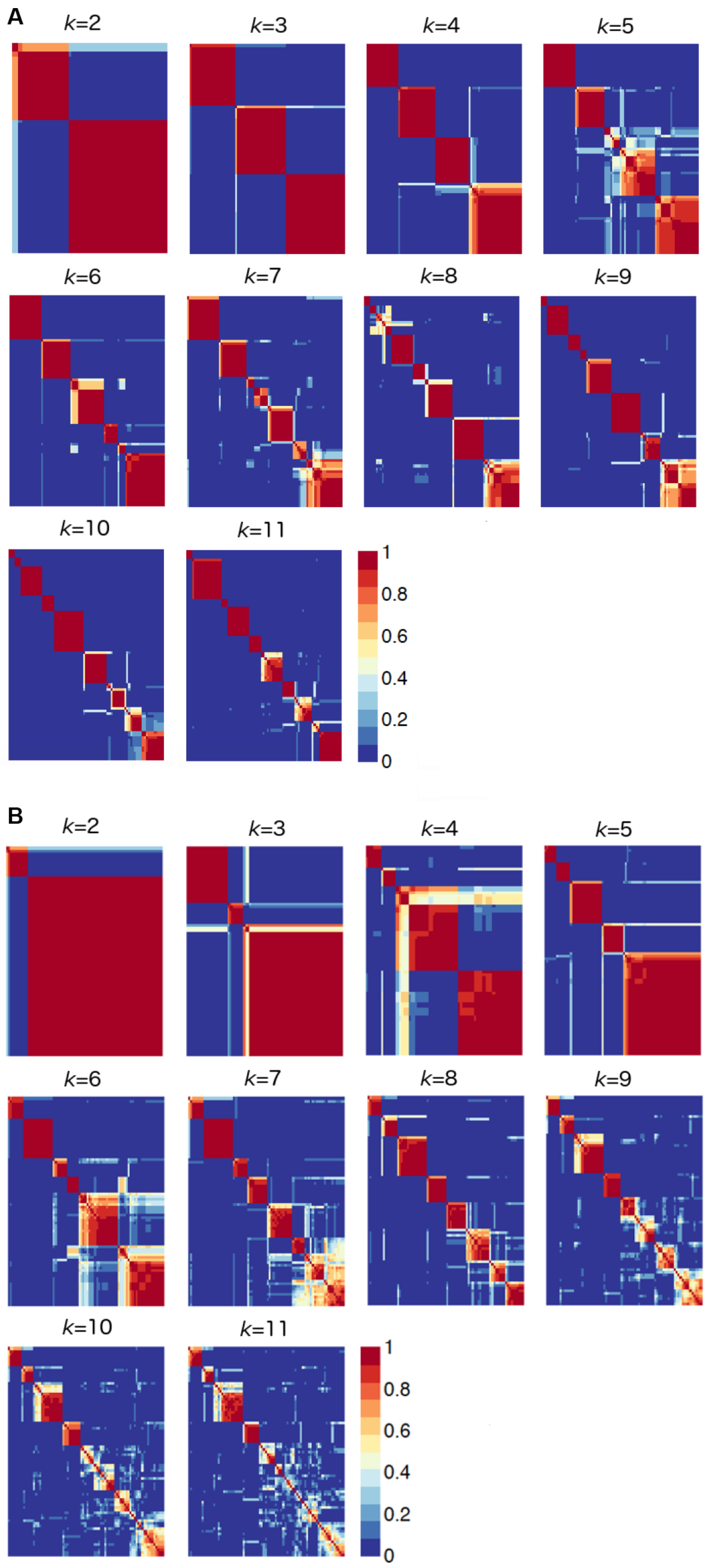

Supplemenatry Figure S9: Rank consensus matrices generated by the R package *NMF* [51]. Consensus matrices computed from 50 runs for each value of rank *k* across (**A**) human and (**B**) mouse datasets.

Supplementary Table S1: PDX model metadata

Supplementary Table S2: Read mapping statistics

Model	Total Reads	Mapped Reads	% Mapped Reads	% Annotated Reads	Human Reads	Mouse Reads	Mouse Reads	% Human
BR0555FJP4M8	62542739	58464484	93.5	44626127	76.3	54671744	3792740	93.5
BR0744F	43437389	41401028	95.3	30039740	72.6	34985536	6415492	84.5
BR0869F	54076262	50599968	93.6	33562118	66.3	48441190	2158778	95.7
BR1126F	61331401	56945968	92.8	43946540	77.2	54442131	2503837	95.6
CN0330F	42693376	40474796	94.8	32299372	79.8	35976942	4497854	88.9
CN0375F	42565436	40409136	94.9	32147764	79.6	30427086	9982050	75.3
CN0446F	43295778	41190206	95.1	29879372	72.5	37992068	3198138	92.2
CN0458F	43311392	41313132	95.4	35918603	86.9	38417607	2895525	93.0
CN1854F	43649301	41694281	95.5	34508067	82.8	39375433	2318848	94.4
CRC040	42759607	40493334	94.7	34413394	85.0	35545398	4947936	87.8
CTC174	70702003	65880202	93.2	48319217	73.3	57737942	8142260	87.6
CTG0159	66421199	62924102	94.7	42872595	68.1	61187720	1736382	97.2
CTG0160	67582966	63975227	94.7	51266285	80.1	60668632	3306595	94.8
CTG0166	55633817	52699831	94.7	36569923	69.4	50542041	2157790	95.9
CTG0198	63317742	57856084	91.4	34199763	59.1	63397883	1363061	97.9
CTG0199	69601722	64760944	93.0	46288020	71.5	51978904	5877180	89.8
EN0346F	66131394	61449455	92.9	40022876	65.1	41524869	19924586	67.6
EN0419F	57137938	53810721	94.2	32503512	60.4	48790735	5019986	90.7
EN129774	43006866	41140673	95.7	29023291	70.5	39784481	1356192	96.7
EPLG7126	69555960	66043153	94.9	50012639	75.7	57102199	8940954	86.5
EPLG7177	56856185	54015965	95.0	38355161	71.0	51616844	2399121	95.6
EPLG7198	62729961	59002212	94.1	44837184	76.0	54077967	4924245	91.7
EPLG7298	70550148	67193782	95.2	43555502	64.8	61272329	5921453	91.2
EPLG7343	63972721	60751734	95.0	45185827	74.4	57156091	3595643	94.1
EPLG7406	71294499	66874289	93.8	48121426	72.0	47856823	19017466	71.6
EPLG7414	64003077	61000135	95.3	46530898	76.3	57370732	3629403	94.1
EPLG7466	58368323	55317110	94.8	46207143	83.5	51022810	4294300	92.2
EPLG7506	71973670	68211555	94.8	48336173	70.9	64518879	3692676	94.6
EPLG7700	59773620	56520609	94.6	44549140	78.8	47550205	8970404	84.1
EPLG7747	66418350	62635401	94.3	47551697	75.9	56587754	6047647	90.3
EPLG7766	62323687	58691417	94.2	46503433	79.2	52494193	6197224	89.4
EPLG7860	63550986	59964219	94.4	47124290	78.6	54861080	5103139	91.5
EPLG7913	62835852	59386797	94.5	46027517	77.5	49696859	9689938	83.7
HBCX1	61661982	57669932	93.5	40282753	69.9	53907991	3761941	93.5
HBCX10	59022129	52998352	89.8	38985898	73.6	49376931	3621421	93.2
HBCX11	59929713	54798007	91.4	36897399	67.3	50558893	4239114	92.3
HBCX14	59522280	55403300	93.1	38387680	69.3	52878283	2525017	95.4
HBCX19	66187356	60618394	91.6	38247737	63.1	47980711	12637683	79.2
HBCX2	64751546	60508745	93.4	44608860	73.7	55833872	4674873	92.3

HBCX24	66247839	61578003	93.0	45730508	74.3	56784105	4793898	92.2
HBCX3	70965395	66361048	93.5	50091710	75.5	59165645	7195403	89.2
HBCX34	64998184	60524905	93.1	38982266	64.4	58495564	2029341	96.6
HBCX6	68785450	64707637	94.1	47060026	72.7	62241075	2466562	96.2
HBCX7	61731418	57226482	92.7	40874786	71.4	42622236	14604246	74.5
HBCX9	65320259	59623365	91.3	36115460	60.6	55732621	3890744	93.5
HBXF002	43713188	41712800	95.4	30332324	72.7	37707869	4004931	90.4
HBXF079	58593485	54789934	93.5	35291685	64.4	50254849	4535085	91.7
HCXF001	43679637	41481005	95.0	30412903	73.3	39649208	1831797	95.6
HCXF034	42844883	40877255	95.4	32894734	80.5	34223741	6653514	83.7
HLKXF101	43731348	41530789	95.0	30733722	74.0	31093355	10437434	74.9
HLXF016	65250082	61439133	94.2	46653378	75.9	54812854	6626279	89.2
HLXF023	58350982	52962821	90.8	35797192	67.6	31227316	21735505	59.0
HLXF033	59080453	55794091	94.4	43308200	77.6	48260726	7533365	86.5
HLXF036	60395877	56872348	94.2	36742213	64.6	51285011	5587337	90.2
HLXF036LN	72040979	67314012	93.4	42110592	62.6	57416725	9897287	85.3
HLXF048	69567315	64904025	93.3	44431828	68.5	42358444	22545581	65.3
HLXF056	54956720	51111497	93.0	36539452	71.5	41631533	9479964	81.5
HLXF093	58292862	54048883	92.7	36382695	67.3	43963270	10085613	81.3
HOXF031	54699449	51946887	95.0	39540714	76.1	42906796	9040091	82.6
HOXF050	51882482	48814070	94.1	30314748	62.1	47449242	1364828	97.2
HOXF060	56409893	50999389	90.4	31920661	62.6	15385308	35614081	30.2
HOXF062	64384424	60487230	93.9	36137540	59.7	55871102	4616128	92.4
HPAXF006	43822652	41879082	95.6	34260421	81.8	36005319	5873763	86.0
HPAXF014LN	42787191	40960511	95.7	29613526	72.3	36247434	4713077	88.5
HPAXF037	43586040	41658374	95.6	34042310	81.7	35073909	6584465	84.2
HPAXF049	43146686	41324116	95.8	34030187	82.3	38214263	3109853	92.5
IC11LC13	57678042	53710269	93.1	37181492	69.2	51546682	2163587	96.0
IC14LC18	64882503	60589294	93.4	41241824	68.1	47419380	13169914	78.3
IC1TEP	58608031	53660221	91.6	42113157	78.5	52058488	1601733	97.0
IC20DAN	65961544	61567205	93.3	50187375	81.5	59315892	2251313	96.3
IC8LC10	59053328	55961113	94.8	43176290	77.2	50719944	5241169	90.6
IC9LC11	53230774	50116881	94.2	38789998	77.4	43597342	6519539	87.0
LG0567F	62706991	58511950	93.3	44101668	75.4	41214402	17297548	70.4
LG0645F	42762441	40873755	95.6	32560894	79.7	39052823	1820932	95.5
ML5LC66	57615978	54379674	94.4	42985577	79.0	44743725	9635949	82.3
OV0529F	42529594	40845451	96.0	34106273	83.5	38188735	2656716	93.5
OV0857F	65559670	61061940	93.1	43465178	71.2	54241232	6820708	88.8
OV2022F	43483298	41638909	95.8	32296953	77.6	38534128	3104781	92.5
SC131	70664722	66074901	93.5	51177669	77.5	58261176	7813725	88.2

Supplementary Table S3: Estimate scores across human and mouse components

Model	Human				Mouse			Human FAP (log_2 FPKM)	Human CSPG4 (log_2 (FPKM)
	Stromal Score	Immune Score	Estimate Score	Tumour purity	Stromal Score	Immune Score	Estimate Score		
BR0555FJP4M8	−3012.51	−2325.15	−5337.66	0.98	1736.61	1827.43	3564.05	0.14	0.77
BR0744F	−1753.99	−1614.69	−3368.67	0.99	1275.35	2353.23	3628.58	0.67	0.61
BR0869F	−2621.33	−1760.83	−4382.16	1.00	1524.74	2004.72	3529.46	0.21	5.58
BR1126F	−3095.05	−2043.58	−5138.64	0.99	1818.82	1879.17	3697.99	0.14	1.54
CN0330F	−3234.90	−1326.99	−4561.89	1.00	1835.21	1799.77	3634.98	0.14	0.14
CN0375F	−3193.39	−1517.77	−4711.16	1.00	1449.86	1451.98	2901.84	0.14	0.16
CN0446F	−2946.76	−1581.40	−4528.16	1.00	1673.52	2538.91	4212.43	4.39	0.45
CN0458F	−3142.55	−1217.75	−4360.30	1.00	1726.43	1816.31	3542.74	0.45	0.25
CN1854F	−3259.15	−1292.52	−4551.67	1.00	1694.60	1763.93	3458.53	0.94	0.28
CRC040	−3282.15	−1467.75	−4749.90	1.00	1250.41	1049.63	2300.04	0.19	0.15
CTC174	−3171.40	−2116.81	−5288.21	0.99	1510.44	1782.41	3292.85	0.21	0.33
CTG0159	−3172.82	−777.17	−3949.99	1.00	1504.86	1580.49	3085.36	0.79	0.22
CTG0160	−3094.28	−1440.38	−4534.66	1.00	1673.21	2356.21	4029.42	0.14	2.04
CTG0166	−3169.02	−2210.23	−5379.25	0.98	1977.37	1760.46	3737.83	0.45	0.85
CTG0198	−2753.28	−2672.75	−5426.02	0.98	1224.25	1574.99	2799.24	0.67	0.3
CTG0199	−3164.53	−2345.85	−5510.38	0.98	1585.78	1682.04	3267.82	0.14	0.18
EN0346F	−2895.52	−2092.59	−4988.11	0.99	1667.95	1264.86	2932.81	0.18	0.17
EN0419F	−2852.74	−1548.92	−4401.66	1.00	1774.12	1639.28	3413.41	1.07	0.5
EN129774	−2893.23	−2082.84	−4976.08	0.99	1979.38	1800.83	3780.21	0.14	0.31
EPLG7126	−2622.01	−1556.35	−4178.36	1.00	1672.88	1770.88	3443.76	0.14	2.02
EPLG7177	−3137.30	−1651.95	−4789.25	1.00	1228.45	1214.91	2443.36	0.14	0.88
EPLG7198	−2901.43	−795.05	−3696.47	1.00	1464.81	2376.23	3841.04	5.08	0.53
EPLG7298	−3116.25	−1787.05	−4903.31	0.99	1749.25	1543.86	3293.11	0.14	1.7
EPLG7343	−3014.24	−1617.09	−4631.33	1.00	1777.90	1808.48	3586.38	0.14	1.99
EPLG7406	−2977.20	−808.50	−3785.70	1.00	1693.40	1787.91	3481.30	0.14	0.18
EPLG7414	−3161.05	−1489.27	−4650.33	1.00	1613.31	1645.90	3259.21	0.41	0.67
EPLG7466	−2768.49	−999.87	−3768.37	1.00	1851.80	2184.00	4035.80	1.26	3.04
EPLG7506	−1885.04	−1018.08	−2903.11	0.98	1650.95	2509.98	4160.92	2.29	3.39
EPLG7700	−3200.24	−930.02	−4130.26	1.00	1595.09	1795.30	3390.39	0.96	0.16
EPLG7747	−2910.24	−1370.55	−4280.79	1.00	1816.67	2018.78	3835.45	0.14	2.26
EPLG7766	−3132.63	−1385.39	−4518.02	1.00	1924.55	2575.44	4500.00	0.14	0.32
EPLG7860	−3111.61	−1276.40	−4388.01	1.00	1691.34	2114.41	3805.75	0.41	0.48
EPLG7913	−3116.31	−1089.61	−4205.92	1.00	1873.58	2156.95	4030.53	0.95	2.73
HBCX1	−2980.83	−1802.89	−4783.71	1.00	1804.46	1717.54	3522.00	0.47	0.97
HBCX10	−2811.91	−1856.38	−4668.29	1.00	1807.88	2044.15	3852.03	0.55	2.82

HBCX11	−2929.68	−2082.33	−5012.01	0.99	1943.58	1737.70	3681.28	0.33	3.47
HBCX14	−3037.39	−1420.52	−4457.91	1.00	1326.18	2517.45	3843.64	0.22	0.31
HBCX19	−2743.84	−1851.63	−4595.48	1.00	1873.27	1400.97	3274.24	0.89	4.85
HBCX2	−3102.81	−1991.04	−5093.85	0.99	1688.40	1937.63	3626.03	0.26	0.19
HBCX24	−2540.81	−1266.68	−3807.48	1.00	1878.60	1736.20	3614.80	2.1	5.77
HBCX3	−3043.28	−1593.98	−4637.26	1.00	1738.68	1996.33	3735.00	0.63	0.62
HBCX34	−3191.87	−1920.87	−5112.74	0.99	1615.89	2111.77	3727.66	0.23	0.52
HBCX6	−2471.13	−1503.71	−3974.83	1.00	1579.25	1757.44	3336.69	2.08	5.79
HBCX7	−3026.01	−1023.01	−4049.03	1.00	1605.43	1467.15	3072.58	0.14	0.56
HBCX9	−2795.88	−1149.70	−3945.57	1.00	1837.78	2090.36	3928.14	2.91	4.2
HBXF002	−2751.83	−1300.56	−4052.39	1.00	1925.31	2272.95	4198.26	0.19	1
HBXF079	−3061.07	−1939.72	−5000.80	0.99	1657.88	1812.07	3469.95	0.61	0.31
HCXF001	−3063.42	−2053.22	−5116.63	0.99	1937.04	1924.55	3861.60	0.18	0.5
HCXF034	−3352.10	−1524.09	−4876.19	0.99	1980.07	1796.24	3776.31	0.14	0.19
HLKXF101	−1816.05	2329.73	513.68	0.78	1698.85	2399.41	4098.26	0.2	0.14
HLXF016	−2954.25	−1044.06	−3998.31	1.00	1556.76	2005.14	3561.90	0.73	0.57
HLXF023	−1883.43	2393.99	510.56	0.78	1143.94	2446.76	3590.69	0.18	0.15
HLXF033	−2964.64	−1494.16	−4458.80	1.00	1573.29	1781.84	3355.13	0.49	0.23
HLXF036	−2828.53	−1429.62	−4258.15	1.00	1730.46	2312.40	4042.85	4.81	0.47
HLXF036LN	−2849.29	−1360.09	−4209.38	1.00	1838.12	2657.09	4495.22	4.72	0.34
HLXF048	−1826.06	2318.13	492.07	0.78	1501.31	2671.34	4172.65	0.26	0.15
HLXF056	−2841.91	−1427.30	−4269.21	1.00	1836.62	2076.67	3913.28	0.17	0.29
HLXF093	−2812.45	−94.31	−2906.76	0.98	1653.95	2204.55	3858.50	1.79	2.06
HOXF031	−1732.65	−1888.33	−3620.99	1.00	1843.92	2128.25	3972.17	1.44	0.95
HOXF050	−2628.26	−2358.77	−4987.03	0.99	1829.36	2305.19	4134.55	0.72	1.66
HOXF060	−2333.61	−1609.68	−3943.28	1.00	1459.40	2325.93	3785.33	0.14	0.2
HOXF062	−1398.51	−1450.09	−2848.60	0.98	1921.85	2099.90	4021.75	1.91	1.42
HPAXF006	−3105.66	−1665.23	−4770.89	1.00	1892.24	1854.91	3747.15	0.2	0.65
HPAXF014LN	−3161.87	−1337.16	−4499.03	1.00	1981.52	2234.51	4216.03	0.42	3.17
HPAXF037	−3036.70	−1367.99	−4404.69	1.00	1748.02	1915.19	3663.21	0.14	1.46
HPAXF049	−3134.56	−1008.60	−4143.16	1.00	1644.54	2434.91	4079.46	0.14	0.28
IC11LC13	−3053.63	−1650.17	−4703.80	1.00	1717.69	2240.75	3958.44	0.25	0.45
IC14LC18	−3047.66	−793.14	−3840.80	1.00	1524.62	2359.91	3884.54	0.25	0.2
IC1TEP	−3210.95	−1709.15	−4920.10	0.99	1647.42	1987.35	3634.78	0.14	4.41
IC20DAN	−2827.91	−1094.26	−3922.17	1.00	1217.22	2325.27	3542.49	1.95	5.05
IC8LC10	−3267.26	−1375.33	−4642.59	1.00	1646.61	2050.15	3696.76	0.18	0.3
IC9LC11	−3271.18	−1632.86	−4904.03	0.99	1699.40	1877.71	3577.11	0.14	0.27
LG0567F	−3207.22	−655.14	−3862.36	1.00	1586.46	1475.91	3062.37	0.23	0.2
LG0645F	−3084.77	−1130.85	−4215.62	1.00	1754.63	2340.22	4094.85	0.76	0.42
ML5LC66	−2786.88	−679.54	−3466.43	1.00	1672.78	2038.08	3710.86	0.79	0.32
OV0529F	−2832.65	−1208.06	−4040.72	1.00	1923.28	2110.24	4033.52	0.25	0.25

OV0857F	−1940.07	−2235.18	−4175.26	1.00	1179.14	1367.43	2546.57	0.18	2.23
OV2022F	−2464.81	−2093.62	−4558.44	1.00	1914.10	2065.22	3979.33	0.33	1.2
SC131	−3334.63	−1989.46	−5324.09	0.98	1745.87	1754.28	3500.15	0.2	0.28

Supplementary Table S4: Human and mouse cluster details

Model	Human (no. of clusters = 9)		Mouse (no. of clusters = 5)	
	Cluster Number	Strength	Cluster Number	Strength
EPLG7766	1	0.95	5	0.15
BR0744F	3	−0.03	5	0.79
CN0446F	4	0.93	5	0.55
HPAXF049	4	0.92	5	0.61
HLKXF101	5	0.98	5	0.60
HLXF048	5	0.99	5	0.73
HOXF060	6	0.76	5	0.75
CTG0160	1	0.94	4	0.19
EPLG7298	1	0.91	4	0.49
EPLG7343	1	0.95	4	0.76
EPLG7506	1	0.68	4	0.37
EPLG7747	1	0.95	4	0.25
EPLG7860	1	0.94	4	−0.53
EPLG7913	1	0.91	4	0.56
HLXF093	1	0.77	4	−0.13
IC11LC13	1	0.94	4	−0.45
IC1TEP	1	0.94	4	0.55
BR0869F	2	0.94	4	0.60
BR1126F	2	0.93	4	0.34
HBCX1	2	0.96	4	0.27
HBCX10	2	0.96	4	0.63
HBCX11	2	0.94	4	0.61
HBCX14	2	0.95	4	0.15
HBCX24	2	0.96	4	0.47
HBCX6	2	0.96	4	0.42
HBCX9	2	0.96	4	0.73
EN0419F	6	−0.04	4	0.30
HOXF050	6	0.84	4	0.64
HOXF062	6	0.84	4	0.59
HBCX2	7	0.82	4	0.74
HBCX34	7	0.97	4	0.75
HBXF079	7	0.96	4	0.54
CTG0166	8	0.96	4	0.52
CTG0198	8	0.97	4	0.55
CTG0199	8	0.98	4	0.62
CTG0159	9	0.51	4	0.66
EPLG7198	9	0.56	4	0.12

EPLG7406	9	0.68	4	0.15
EPLG7414	9	0.59	4	0.49
EPLG7466	9	0.05	4	0.70
EPLG7700	9	0.49	4	−0.28
HBCX7	9	−0.20	4	0.70
HLXF016	9	0.05	4	−0.13
HLXF033	9	0.55	4	0.66
HLXF036	9	0.15	4	0.69
HLXF036LN	9	0.45	4	0.26
HLXF056	9	0.69	4	0.59
EPLG7126	1	0.95	3	0.67
CRC040	4	0.92	3	0.73
HLXF023	5	0.99	3	−0.22
OV0857F	6	0.85	3	0.84
CTC174	7	0.97	3	0.79
LG0567F	9	0.59	3	0.25
HBXF002	2	0.78	2	0.93
LG0645F	3	−0.25	2	0.77
OV0529F	3	0.51	2	0.80
CN0330F	4	0.93	2	0.90
CN0375F	4	0.93	2	0.93
CN0458F	4	0.93	2	0.78
CN1854F	4	0.93	2	0.94
EN0346F	4	0.18	2	0.27
HCXF001	4	0.56	2	0.93
HCXF034	4	0.94	2	0.92
HPAXF006	4	0.93	2	0.93
HPAXF014LN	4	0.93	2	0.92
HPAXF037	4	0.94	2	0.86
EN129774	6	0.40	2	0.94
HOXF031	6	0.63	2	0.89
OV2022F	6	0.80	2	0.92
EPLG7177	1	0.86	1	0.75
IC20DAN	1	0.93	1	0.81
IC9LC11	1	0.93	1	0.91
HBCX19	2	0.90	1	0.74
BR0555FJP4M8	7	0.95	1	0.68
HBCX3	7	0.96	1	0.77
IC14LC18	9	0.39	1	0.84
IC8LC10	9	0.64	1	0.91
ML5LC66	9	0.23	1	0.88
SC131	9	0.16	1	0.91

Supplementary Table S5: (a) Human and (b) mouse cluster metagenes

Supplementary Table S6: Mouse cluster functional enrichment (functions achieving significant enrichment at benjamini p < 0.01)

Supplementary Table S7: Gene expression correlation between human CDH1 and mouse genes

Supplementary Table S8: Genes with highest number of inter-species connections in human-mouse expression stringent (|r| > 0.85) correlation network

Gene symbol	Ensembl ID	No. of connected nodes	
		Human	Mouse
**(a) Human genes**			
MIF	ENSG00000240972	58	17
SCAND1	ENSG00000171222	35	10
AC119673.1	ENSG00000268313	0	6
TMEM160	ENSG00000130748	38	3
POLR2L	ENSG00000177700	97	2
AC040977.1	ENSG00000269871	4	2
PRR7	ENSG00000131188	48	1
MZT2B	ENSG00000152082	46	1
MZT2A	ENSG00000173272	62	1
TRAPPC5	ENSG00000181029	81	1
**(b) Mouse genes**			
Ddx6	ENSMUSG00000032097	8	33
Hnrnpa2b1	ENSMUSG00000004980	3	60
Ddx3x	ENSMUSG00000000787	2	26
Chmp2b	ENSMUSG00000004843	2	34
Actr2	ENSMUSG00000020152	2	43
Tm9sf3	ENSMUSG00000025016	2	30
Dcun1d5	ENSMUSG00000032002	2	27
Cmtm6	ENSMUSG00000032434	2	18
Dync1li2	ENSMUSG00000035770	2	22
Fam168b	ENSMUSG00000037503	2	22
Bzw1	ENSMUSG00000051223	2	41
Hnrnpa3	ENSMUSG00000059005	2	43
Flii	ENSMUSG00000002812	1	40
Xpo1	ENSMUSG00000020290	1	42
Tmx1	ENSMUSG00000021072	1	29
Snrpc	ENSMUSG00000024217	1	39

Cct8	ENSMUSG00000025613	1	38
Adh5	ENSMUSG00000028138	1	16
Strap	ENSMUSG00000030224	1	18
Rnf126	ENSMUSG00000035890	1	43
Ubald1	ENSMUSG00000039568	1	39
Shfm1	ENSMUSG00000042541	1	45
Hnrnph2	ENSMUSG00000045427	1	33
Scand1	ENSMUSG00000046229	1	61
H2afj	ENSMUSG00000060032	1	55

Supplementary Table S9: PDX samples (human component) predicted to be hypoxic and normoxic based on human *MIF* and mouse *Ddx6* expression

Sample	Human MIF/mouse Ddx6 expression level	Predicted status
BR0744F	Low/High	Normoxic
BR0869F	High/Low	Hypoxic
CN0330F	Low/High	Normoxic
CN0375F	Low/High	Normoxic
CN0446F	Low/High	Normoxic
CRC040	Low/High	Normoxic
CTG0159	High/Low	Hypoxic
CTG0198	High/Low	Hypoxic
EN129774	Low/High	Normoxic
EPLG7506	High/Low	Hypoxic
HBCX14	High/Low	Hypoxic
HBCX2	High/Low	Hypoxic
HBCX34	High/Low	Hypoxic
HBXF002	Low/High	Normoxic
HCXF001	Low/High	Normoxic
HCXF034	Low/High	Normoxic
HLKXF101	Low/High	Normoxic
HOXF31	Low/High	Normoxic
HOXF60	Low/High	Normoxic
HPAXF006	Low/High	Normoxic
HPAXF014LN	Low/High	Normoxic
HPAXF037	Low/High	Normoxic

HPAXF049	Low/High	Normoxic
IC1TEP	High/Low	Hypoxic
IC20DAN	High/Low	Hypoxic
OV2022F	Low/High	Normoxic

Supplementary Table S10: Mouse genes differentially expressed between basal triple negative and luminal-B ER+ breast cancer PDX samples

GENE	ENSEMBL ID	Human Gene Name	Mean Expression	Log2 FC	*p*-value	FDR
Hmga1-rs1	ENSMUSG00000078249	NA	26.88	4.07	4.26E−6	2.43E−3
Serpinb2	ENSMUSG00000062345	SERPINB2	181.89	3.32	1.35E−4	3.18E−2
Arg1	ENSMUSG00000019987	ARG1	1222.78	3.16	6.94E−6	3.70E−3
Esm1	ENSMUSG00000042379	ESM1	187.10	2.14	3.20E−6	2.13E−3
Mmp12	ENSMUSG00000049723	MMP12	1037.65	2.00	8.40E−5	2.43E−2
Adam8	ENSMUSG00000025473	ADAM8	1337.67	1.87	5.75E−5	2.09E−2
Nos2	ENSMUSG00000020826	NOS2	305.04	1.82	1.69E−6	1.50E−3
Igfbp3	ENSMUSG00000020427	IGFBP3	7506.89	1.81	3.22E−13	1.29E−9
Sh2d5	ENSMUSG00000045349	SH2D5	146.91	1.74	4.35E−5	1.74E−2
Pgf	ENSMUSG00000004791	PGF	228.44	1.61	2.52E−4	4.91E−2
Spint2	ENSMUSG00000074227	SPINT2	68.12	−1.82	1.25E−4	3.04E−2
Tril	ENSMUSG00000043496	TRIL	192.98	−1.93	1.02E−8	1.38E−5
St6galnac2	ENSMUSG00000057286	ST6GALNAC2	98.93	−2.32	3.63E−5	1.61E−2
Egflam	ENSMUSG00000042961	EGFLAM	119.44	−2.96	8.18E−8	9.34E−5
Epcam	ENSMUSG00000045394	EPCAM	4.44	−3.03	1.94E−4	4.07E−2
Ltf	ENSMUSG00000032496	LTF	54.78	−3.34	2.04E−6	1.63E−3
Ggt1	ENSMUSG00000006345	GGT1	17.29	−3.53	5.82E−5	2.09E−2
Csn1s1	ENSMUSG00000070702	CSN1S1	6.02	−4.49	3.09E−6	2.13E−3

Supplementary Table S11: List of (a) PDX, (b) UBCS, (c) TCGA and (d) CCLE clinical samples used in basal triple negative breast cancer case study

Supplementary Table S12: Genes differentially expressed between triple negative and ER+ breast cancer in clinical stroma (Finak et al. 2008)

ProbeName	GeneName	Genbank ID	log2 FC	*p*-value	FDR
A_32_P184464	ROPN1	NM_017578	4.11	3.86E−18	1.58E−13
A_23_P340698	MMP12	NM_002426	2.83	4.59E−13	3.14E−9
A_23_P4714	MIA	NM_006533	2.62	9.35E−12	4.27E−8

A_23_P78248	KRT23	NM_173213	2.43	2.53E−10	7.43E−7
A_23_P167159	SCRG1	NM_007281	2.33	3.07E−8	5.48E−5
A_24_P114249	GALNT3	NM_004482	2.05	2.46E−4	4.39E−2
A_23_P39955	ACTG2	NM_001615	1.96	7.88E−7	8.98E−4
A_23_P362694	C4orf7	NM_152997	1.89	1.60E−5	7.16E−3
A_23_P91230	SLPI	NM_003064	1.89	1.29E−9	3.30E−6
A_23_P348146	FLJ30046	NM_144595	1.87	7.05E−6	3.86E−3
A_23_P107911	KLK10	NM_002776	1.85	1.98E−6	1.63E−3
A_23_P1691	MMP1	NM_002421	1.78	9.57E−9	1.79E−5
A_23_P253123	VGLL1	NM_016267	1.72	1.04E−14	1.07E−10
A_24_P297539	UBE2C	NM_181803	1.69	2.88E−5	1.08E−2
A_23_P357207	C6orf117	NM_138409	1.68	1.74E−5	7.59E−3
A_23_P5301	TFCP2L1	NM_014553	1.66	1.25E−5	5.83E−3
A_32_P216520	WIF1	NM_007191	1.64	1.88E−4	3.74E−2
A_23_P134176	SOD2	NM_000636	1.62	5.87E−6	3.40E−3
A_23_P76249	KRT6B	NM_005555	1.53	1.68E−7	2.37E−4
A_23_P214950	PERP	NM_022121	1.52	1.12E−6	1.17E−3
A_24_P104407	DMN	NM_145728	1.51	5.50E−6	3.24E−3
A_23_P31073	MYB	NM_005375	−1.60	9.32E−5	2.45E−2
A_24_P347431	FOXA1	NM_004496	−1.60	1.17E−9	3.20E−6
A_23_P372234	CA12	NM_001218	−1.63	4.31E−5	1.46E−2
A_23_P50167	SLC39A6	NM_012319	−1.69	3.71E−5	1.30E−2
A_23_P37127	FOXA1	NM_004496	−1.85	2.56E−11	1.05E−7
A_23_P393099	TFF3	NM_003226	−2.20	7.04E−5	2.13E−2
A_23_P113111	AR	NM_000044	−2.35	3.73E−6	2.60E−3
A_23_P309739	ESR1	NM_000125	−2.46	3.86E−6	2.64E−3

Supplementary Table S13: (a) Stromal, (b) epithelial-mesenchymal transition (EMT), (c) cancer stem cell (CSC) and (d) reactive stroma signatures used in this study

(a) Yoshihara et al. 2013		(b) Loboda et al. 2011		(c) Creighton et al. 2009		(d) Farmer et al. 2008
**Stromal**	**Immune**	**EMT Up**	**EMT Down**	**CSC Up**	**CSC Down**	**Reactive stroma**
BTK	FGR	TBXA2R	HS3ST1	CPS1	SEMA3F	DCN
FMO1	ITGAL	CLDN11	TTC22	VIM	BAIAP2L1	CSPG2
DCN	IL32	FHL1	SYT7	POU2F2	TRAPPC6A	CDH11
IGF1	TRAF3IP3	VIM	ACPP	TRIO	CYB561	COL3A1
HGF	TYROBP	DKK3	SH3YL1	ARID1B	ANLN	FAP

GPR124	CD74	SLC2A3	CDH1	FSTL4	POLA2	PEDF
MSR1	TNFRSF1B	EML1	EPN3	PSD	IL20RA	FBN1
PTGER3	LCP2	NAV3	PRSS8	HIPK2	EPN3	PDGFRL
FCGR2B	RAB27A	CYBRD1	LAMA3	TRAM2	NEDD4L	CTSK
APBB1IP	ARHGAP15	FGFR1	LAMC2	PDE4A	KIF1B	HTRA1
FAP	IL4R	EPDR1	CDH3	MEF2A	ERBB3	ASPN
ITM2A	CST7	MMP2	ATP2C2	ATP2B1	GSTO2	SPARC
RUNX1T1	PTPRC	LEPREL1	ERBB3	MAP4K4	FGFR2	COL5A2
SP140	FYB	TGFB2	PLA2G10	ZCCHC6	ROGDI	LOXL1
COL5A3	GMIP	LGALS1	DAPP1	FXYD5	KIAA1107	MMP2
NOX4	RGS1	TTC28	MBNL3	PLEKHG2	NFATC3	SPON1
ARHGAP28	LYZ	SRPX	GRHL2	PTGS1	MCM2	SFRP4
SIGLEC1	NCF4	TRPA1	CEACAM6	NUP188	MAP3K13	ITGBL1
HEPH	IL2RB	SYDE1	TMPRSS11E	FKBP5	NTN4	CALD1
CD200	GZMH	CLIP3	SLC6A14	TNRC6B	TPD52	COPZ2
MXRA5	GZMB	DFNA5	FXYD3	KPNA3	EDN1	MFAP2
ZNF423	NFKBIA	SERPINE1	BLNK	DNM1	MAGI3	ANGPTL2
FOXF1	RASSF2	CCL2	DSP	HPS1	SERTAD4	PLAU
ATP8B4	HCK	PMP22	WFDC2	ZBTB16	ULK2	COL1A2
WISP1	SAMHD1	WNT5B	LIPG	SOX6	GRHL2	LRRC17
CD33	CORO1A	COL12A1	FA2H	SH2B3	KIF9	C1QTNF3
COMP	CD37	RBM24	TOX3	QKI	BIRC5	SNAI2
TFEC	LILRB1	CAP2	TMC5	GGCX	KIF4A	PCOLCE
WNT2	TGFB1	BVES	MYH14	ST3GAL5	EPB41L4B	POSTN
SFRP4	NKG7	LAMA4	CEACAM5	ARHGEF2	CRISP3	ECM2
RARRES2	LPXN	LOX	INPP4B	TMED5	TSPAN15	FBLN1
ASPN	IL10RA	SPARC	SCNN1A	CNR1	RASSF7	ADAM12
ECM2	TCIRG1	DPYSL3	ARHGDIB	KLF12	PLCB4	MMP11
CXCL12	CD69	GNB4	ITGB6	CCND2	PIN4	AEBP1
MS4A6A	CLEC2B	ADAM23	GALNT3	CCDC92	TRPS1	PDGFRB
MS4A4A	SELPLG	EPB41L5	TSPAN1	ZMYM2	MCM4	GAS1
LY86	BIN2	WIPF1	IRF6	SLC25A16	TJP3	COL6A3
PCDH12	SH2B3	NID1	PPL	CD97	ZNF85	RARRES2
PDGFRB	ARHGDIB	PLAGL1	BSPRY	IRF5	TSPAN12	COL6A1
CD86	CLEC4A	CTGF	GJB6	PTPRA	COBL	TGFB3
IL18R1	KLRB1	RECK	SLPI	BTG1	AGR2	NDN
RGS4	VNN2	SRGN	SH2D3A	DDB2	ELAVL2	C1R
FASLG	GZMK	BICC1	OVOL2	DTX1	SHB	LRP1
TNFSF4	ITK	IL13RA2	STEAP4	NT5E	GATA3	COL10A1
LPPR4	ZAP70	PTGIS	AP1M2	BIN1	SLC9A3R1	DPYSL3
TCF21	ITGA4	EVI2A	KLK10	ARHGAP24	INPP4B	OLFML2B
TNN	GNLY	ARMCX1	KLK8	FRAS1	CCDC34	MMP14
KCNJ8	IL18RAP	TNFRSF19	RAB25	PPP3CA	FBXO3	DACT1
RAMP3	WIPF1	GNG11	ALDH3B2	TCHP	EXPH5	MGC3047
ITIH5	PLEK	CPA4	SPINK5	DYM	PPM1H	THBS2
COL10A1	NCF2	CHN1	TTC9	IFNAR1	FRK	

PTGIS	CD2	ATP8B3	VTCN1	EMP3	GCNT2	
F13A1	CD48	COL5A1	GRHL1	RERE	CAP2	
IL1B	GBP1	AKAP12	HOOK1	SH3BGRL3	PRLR	
CD93	TNFAIP3	RFTN1	DSC2	SSBP2	WWC1	
KDR	MFSD1	GFPT2	EHF	SH3KBP1	STC2	
ISLR	ADCY7	MAP1B	ELF5	CHST7	AMOTL2	
AOC3	CSTA	POPDC3	MAP7	ARID5B	HES1	
RSAD2	SRGN	POSTN	SCEL	BICD1	C3orf52	
PLXNC1	NCKAP1L	DOCK10	LCP1	ST3GAL2	CISH	
ENPP2	PTGER2	ANGPTL2	RAPGEF5	FMNL2	PLCH1	
TLR2	CCR7	TTLL7	IL1RN	ABR	EPB41L5	
LRRC32	FGL2	AOX1	FGFBP1	GATS	CENPA	
SULF1	TPST2	FGF5	TMPRSS4	CYGB	GRB14	
CH25H	RAC2	FGF2	TRIM29	ANTXR2	KIAA1324	
CILP	ARHGEF6	GLIPR1	POU2F3	SMPD1	SSX2IP	
CXCL9	LSP1	CMTM3	TMPRSS13	NDST2	PIK3R3	
ENPEP	GMFG	CDH11	PAK6	CTNNB1	IRF6	
LUM	LILRB2	COL6A1	GPR87	DDIT4	NEK2	
HDC	PTPRE	EMP3	RASGEF1B	LUZP1	CENPF	
IGSF6	ALOX5AP	SLC47A1	ERP27	KLF13	WDR34	
PIK3R5	GIMAP6	ATP8B2	GCNT3	NFRKB	BSPRY	
CXCL14	GIMAP4	AFF3	MARVELD3	CHST11	LRP11	
ADAM12	DOCK2	RBMS3	CNKSR1	EIF1	KIAA1217	
SERPING1	MICAL1	TMEM47	PVRL4	CHST2	PLS1	
MMP3	AOAH	ZEB1	CGN	BASP1	IFT81	
EDNRA	HLA-F	ANKRD1	ANXA9	FLII	WWP1	
SPON1	CASP1	LIX1L	S100A8	PDE4DIP	LPGAT1	
RASGRP3	CD27	DAB2	ILDR1	PER1	PARD6B	
ABCA6	GPR65	HS3ST3A1	FGD2	POLR2A	CSE1L	
ADAMTS5	PSTPIP1	AK5	MAL2	MAML2	SPDEF	
SAMSN1	BCL2A1	UCHL1	FAM83A	EIF4ENIF1	OVOL2	
VSIG4	IRF8	ADAMTS1	LCN2	SMYD3	PODXL	
ERG	VAV1	NRG1	C11orf52	ZNF335	MTX2	
SPON2	EMP3	ST3GAL2	ST14	APOBEC3G	MYO5C	
COX7A1	MYO1F	MRAS	MPP7	MYCBP2	THAP10	
PLXDC1	CD53	NEXN	ANK3	CACNA1G	FOXA1	
ITIH3	S100A8	FSTL1	TMEM45B	GAS7	TULP4	
VCAM1	MSN	IGFBP7	ANKRD22	FYN	MLLT4	
DDR2	IL2RG	PTX3	MARVELD2	SLC11A1	GINS2	
OLFML2B	ADAM8	EDIL3	GPR110		TOP2A	
FRZB	FLI1	COL1A2	SORBS2		RHPN2	
ACTG2	RABGAP1L	DLC1	PROM2		RAB25	
COL6A3	LY96	HTRA1	MAPK13		MTL5	
LMOD1	SLA	GPR176	KRTCAP3		INADL	
TRAT1	FCER1G	JAM3	DMKN		TSPAN2	
FBLN2	ITGB2	FBN1	CCDC64B		VAV3	

CCR1	CCL5	TUB	DENND2D		TMEM106C	
EMCN	LAPTM5	C16orf45	FAM84A		GRHL1	
EDIL3	GBP2	RBPMS2	SPRR3		DSC2	
COL1A2	CTSS	GREM1	S100A9		PRRG4	
GREM1	S100A9	TUBA1A	C1orf106		MAP7	
COL3A1	MNDA	AXL	ELF3		PKIB	
ZEB2	CYBB	COL3A1	CDS1		KCNK1	
LDB2	TPP1	ZEB2	S100P		STX6	
ITGAM	CD3D	ANTXR1	SYK		BZW2	
ZFPM2	LAIR1	ZFPM2	RASEF		KCTD3	
CD14	CD300A	CDH2	FBP1		LRRC1	
SGCD	HLA-DPA1	HAS2	AQP3		FNBP1L	
FPR1	TAP1	PDE7B	FAAH2		ARHGAP29	
COL8A2	RHOH	NEGR1	SPINT1		MNS1	
C3AR1	IL7R	BNC2	WDR72		SHROOM3	
LRRC15	LGALS9	SUSD5	MYO5B		LNX2	
CLEC7A	RNASE6	HEG1	TMC4		MARVELD3	
C1QB	CD52	MSRB3	SPINT2		TOM1L1	
C1QA	PTGER4	BDNF	KLK5		ZNF750	
CD248	GLRX	PTRF	KLK6		PTPRF	
SCUBE2	P2RY14	CDH4	FAM84B		IGSF3	
CD163	RHOG	F2R	NPNT		ATP1B1	
MAF	HCLS1	AP1S2	PTAFR		CGN	
CDH5	PRF1	C1S	SPRR1B		ENSA	
BGN	LCK	NDN	SPRR1A		OAZ3	
CSF1R	IKZF1	PAPPA	HS6ST2		VASH2	
PAPPA	EVI2B	GPC6	FUT3		CREB3L4	
OLFML1	SELL	FAM101B	KRT19		NVL	
SH2D1A	CCDC69	LHFP	AGR3		PKP4	
PRKG1	CD247	KIRREL	CNTNAP2		EIF2B5	
THBS2	MAFB	PRKD1	GOLT1A		PLK2	
PDE2A	HLA-DMA	PRR16	FUT1		CASK	
COL14A1	HLA-DRA	FLRT2	GRAMD2		MAL2	
TLR7	LST1	NAP1L3	GPX2		SLC39A4	
MFAP5	LTB	COL4A1	PRR15		PARD3	
ITGBL1	HLA-E	ZNF788	MUC20		MAGI1	
EGFL6	HLA-G	FAT4	STX19		SUV39H2	
COL15A1	HLA-B	NUDT11	TMEM125		MARVELD2	
AIF1	PTPRCAP	TCF4	B3GNT3		CNKSR3	
DIO2	PVRIG	ANXA6	OR2A4		ENAH	
	HLA-DPB1	ELOVL2	CLDN7		SH3RF1	
	IFI30	SIRPA	TMEM30B		SORBS2	
		ALPK2	C1orf116		USP43	
		COL5A2	PKP3		PROM2	
		DIO2	FAM110C		KRTCAP3	
		PNMA2	KRT5		PPP1R9A	

		TMEM158	KRT16		F11R		
			CLDN4		TMPRSS3		
			S100A14		RDH13		
			LAMB3		SHANK2		
			MB		NFIA		
			PPP1R14C		BPNT1		
			SERPINB5		CAPN13		
			CTAGE4		SLC16A14		
			EPPK1		PDLIM5		
			CKMT1B		ELF3		
			ARHGAP8		CDS1		
			C1orf210		TM4SF18		
			CD24		RPL39L		
					CDC25A		
					KIAA0895		
					PTTG1		
					CHMP4C		
					COX6C		
					RASEF		
					NKX3-1		
					SPINT2		
					TK1		
					DEGS2		
					FAM84B		
					NPNT		
					EFNA1		
					B3GALNT1		
					PGM2		
					TM4SF1		
					SMAD1		
					GPR37		
					FRMD3		
					MRPL13		
					CBX2		
					CTBP2		
					ZNF77		
					C1orf172		
					IRX5		
					C8orf4		
					PPFIA3		
					PAWR		
					IRX3		
					MLF1		
					TSPYL5		
					GPR137C		
					SNRPE		

					IDH2	
					TMEM30B	
					EPHB3	
					ZNF93	
					CDCA2	
					JAG2	
					PBX1	
					GCNT1	
					BCAM	
					HN1	
					MYO6	
					SLC22A5	
					VEPH1	
					IPP	
					MB	
					PAX9	
					FANK1	
					TCEA3	
					CFB	
					LGR4	
					PPAPDC2	
					GTF2H5	

EMT = Epithelial-mesenchymal transition
CSC = Cancer stem cell.

Supplementary Table S14: Hyper-geometric *p*-values generated as a result of overlap between cell type signaures and breast cancer case study differentially expressed gene lists

Signature	BTNBC				ER+ luminal-B			
	PDX (1127[a])	PDX FAP/ CSPG4 low (793[a])	TCGA (1368a)	TCGA low stroma (1763[a])	PDX (511[a])	PDX FAP/ CSPG4 low (569[a])	TCGA (876[a])	TCGA low stroma (847[a])
**Stromal (137[b])**	6.00E−4	3.30E−3	1	0.94	0.94	0.36	0.88	0.59
**CSC Up (90[c])**	1.60E−4	1.59E−3	0.79	1.83E−2	0.55	0.4	0.96	0.96
**CSC Down (211[c])**	0.53	0.25	0.87	0.86	1.26E−3	8.66E−3	1.29E−5	7.65E−7
**EMT Up (144[d])**	7.77E−16	4.17E−10	6.63E−2	2.05E−7	0.31	5.38E−3	0.39	8.05E−2
**EMT Down (156[d])**	1.20E−7	2.68E−6	9.20E−8	7.29E−5	6.31E−2	0.32	4.21E−5	2.54E−5
**Reactive stroma (50[e])**	6.08E−4	3.04E−3	1.00	4.82E−1	1.00	1.00	0.94	0.94

[a]Number of genes over-expressed in TNBC or ER+ luminal-B.
[b]From Yoshihara et al. 2013.
[c]Breast cancer stem cell (CSC) signature from Creighton et al. 2009.
[d]Epithelial-mesenchymal transition (EMT) signature from Loboda et al. 2011.
[e]From Farmer et al. 2008.
